# Unconventional C—Hlg···H–C (Hlg = Cl, Br, and I) Interactions Involving Organic Halides: A Theoretical Study

**DOI:** 10.3390/molecules29235606

**Published:** 2024-11-27

**Authors:** Sergi Burguera, Antonio Bauzá

**Affiliations:** Department of Chemistry, Universitat de les Illes Balears, Ctra. de Valldemossa km 7.5, 07122 Palma, Baleares, Spain

**Keywords:** organic halides, noncovalent interactions, computational study

## Abstract

In this study, unconventional C—Hlg···H–C (Hlg = Cl, Br, and I) interactions involving sp, sp^2^, and sp^3^ organic halides were investigated at the RI-MP2/aug-cc-pVQZ level of theory. Energy Decomposition Analyses (EDA) and Natural Bonding Orbital (NBO) studies showed that these intermolecular contacts are mainly supported by orbital and dispersion contributions, which counteracted the unfavorable/slightly favorable electrostatics due to the halogen–hydrogen σ-hole facing. In addition, the Bader’s Quantum Theory of Atoms in Molecules (QTAIM) and the Noncovalent Interaction plot (NCIplot) visual index methodologies were used to further characterize the interactions discussed herein. We expect that the results reported herein will be useful in the fields of supramolecular chemistry, crystal engineering, and rational drug design, where the fine tuning of noncovalent interactions is crucial to achieve molecular recognition or a specific solid-state architecture.

## 1. Introduction

Supramolecular chemists rely on a deep comprehension of noncovalent forces, which are the pillars of modern chemistry [1]. In fact, a proper understanding and intelligent utilization of them is essential to achieve progress in fields such as supramolecular chemistry [2], molecular recognition [3], and materials science [4]. For instance, interactions involving aromatic rings (i.e., π–π stacking [5], cation–π [6], anion–π [7], lp–π [8], and C–H/π [9] interactions) are extremely important in many chemical and biological processes, including molecular sensing [10], crystal engineering [11], and enzymatic mechanisms [12]. In this context, one of the best-known supramolecular forces that is ubiquitous in many chemical and biological systems is hydrogen bonding [13], due to its important roles in molecular recognition, crystal engineering, and chemical reactions [14]. Consequently, a series of studies using the CSD (Cambridge Structural Database) have been carried out to shed light on the impact of this interaction on crystal formation [15]. Related to this, hydrogen bonding interactions [16,17,18] are further classified into strong (for instance, O–H···O or F–H···F, with interaction energies larger than 40 kJ/mol), normal (X–H···A, with interaction energies of up to 20 kJ/mol), and non-classical (X–H···M, X–H···H–M, and X–H···H–B) hydrogen bonds [19,20]. Particularly, these unconventional interactions have bond strengths of 15–25 kJ/mol and H···H distances ranging between 1.7 and 1.9 Å and involve a typical proton donating bond, O–H or N–H, and an unconventional proton acceptor.

In this context, Richardson and coworkers [21] and later Crabtree and collaborators [22,23] reserved the dihydrogen bond (DHB) term for O–H···H–M and N–H···H–M interactions (where M designates a transition metal or boron). Crabtree and coworkers proposed that it is the σ-bond that plays the role of an electron donor for dihydrogen bonded systems [24], introducing the concept of an A–H···σ hydrogen bond, where A–H is the proton-donating bond (Lewis acid) and σ-electrons (Lewis base) are the proton acceptor. Furthermore, C–H···H–C contacts have been considered interactions of this type [25,26]. Closely related, Echeverría and coworkers revealed interesting trends associated with the crystal structure packing and thermal behavior of homonuclear dihydrogen interactions in dimers of linear, branched, and polyhedral alkanes [27]. They also concluded that for numerous H···H links, the interaction energy exceeds 40 or even 80 kJ/mol. Therefore, the importance of these non-classical hydrogen bonding interactions has been demonstrated in numerous processes, such as hydrogen storage [28], control of reactivity and selectivity in solution, hydride reduction, or ligand attachment to transition metal clusters, as well as in proton transfer and separation of gaseous hydrogen [29].

As reported by Robinson and coworkers [30], there are strong shape similarities among the solid-state structures of bromo- and fluoro-subtituted ethynilbenzenes, as determined by single crystal X-ray diffraction, including the interchangeable character between the halogen and ethynyl substituents on these 1,4- and 1,3,5-substituted benzene rings. Taking advantage of that shape complementarity, Wilcken and coworkers have investigated both computationally and experimentally whether an ethynyl moiety is a suitable bioisostere to replace iodine in ligands that form halogen bonds with the protein backbone [31]. Due to its clear similarities, it is intriguing whether these moieties could be involved in the formation of unconventional halogen···hydrogen interactions, thus pointing the halogen σ-hole towards the hydrogen atom of a H-C bond, resembling the A–H···σ hydrogen bond concept introduced by Crabtree and coworkers.

**Figure 1:**
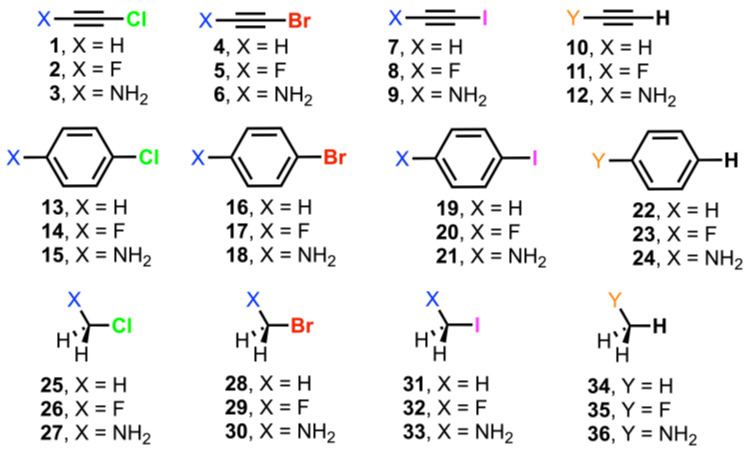
Compounds **1** to **36** used in this study.

In this work, we have performed a computational study to evaluate the ability of sp, sp^2^, and sp^3^ organic halides to establish unconventional C–Hlg···H—C interactions (Hlg = Cl, Br, and I). In more detail, we have used chlorine, bromine, and iodine as halogens and ethyne, benzene, and methane as carbon sp, sp^2^, and sp^3^ moieties, respectively (see Figure 1 below). In addition, we have analyzed the substitution effects by attaching –F, –NH_2_, and H groups to both interacting moieties. Furthermore, Energy Decomposition Analysis (EDA), Natural Bonding Orbital (NBO), Bader’s Quantum Theory of Atoms in Molecules (QTAIM), and Noncovalent Interaction plot (NCIplot) methodologies have been performed in order to further analyze and characterize the physical nature of the interactions studied herein. We believe the results reported here will be useful in the fields of supramolecular chemistry, crystal engineering, and rational drug design, where the fine tuning of noncovalent interactions is crucial to achieve molecular recognition or a specific solid-state architecture.

## 2. Results and Discussion

### 2.1. MEP Analysis

As a preliminary study, we have computed the MEP (molecular electrostatic potential) surfaces of Compounds **1** to **36** (see Table 1) at the RI-MP2/aug-cc-pVQZ level of theory. We used the RI-MP2/aug-cc-pVQZ level of theory since it combines both a robust methodology (MP2) with a quadruple zeta basis set, which is enough to properly describe our system. In addition, the use of the RI approach (which stands for resolution of the identity) allowed us to increase the performance of the MP2 method without losing accuracy during the process. As noted, in all the cases, a positive potential region located at the outermost region of the Hlg and H atoms was found along the vector of the C–Hlg and C–H covalent bonds, commonly known as a σ-hole.

Firstly, if the same type of C hybridization was considered, we observed that the magnitude of the Hlg σ-hole increased from Cl to I (e.g., among Compounds **1**, **4**, and **7** involving sp C atoms or among Compounds **14**, **17**, and **20** involving sp^2^ C atoms), in line with the halogen polarizability values [32]. On the other hand, if the same Hlg atom was considered, we observed that the MEP values became less positive from sp to sp^3^ C hybridization (e.g., Compounds **1**–**3**, **13**–**15**, and **25** to **27**), leading to negative σ-hole MEP values for some compounds (**27**, **30**, and **33**). Additionally, the inclusion of substituents also enhanced or diminished the H/Hlg σ-hole donor ability, as can be deduced by comparing the σ-hole MEP values of Compounds **1** to **3** involving Cl or Compounds **16** to **18** involving Br. In all the cases, the value of the σ-hole was increased (became more positive) by attaching an electron-withdrawing group (–F) or diminished (became less positive) by the presence of an electron-donating group (–NH_2_), owing to the strong electron acceptor/donor properties of each of these two substituents, in agreement with the results obtained by some of us in an earlier work [33]. On the other hand, if no substituent was attached to the organic moiety (–H), we obtained a Hlg/H σ-hole MEP value in between the –F and –NH_2_ substituents, as was expected. Lastly, in the case of the H atom moieties (Compounds **10**–**12**, **22**–**24**, and **34**–**36**), we observed positive electrostatic potential values in all the cases, following the same tendency as that of the halogenated moieties discussed above.

Owing to the presence of a σ-hole in both the Hlg- and H-containing moieties, an electrostatic repulsion is expected to appear upon interacting with each other, thus disfavoring supramolecular complex formation. Thus, we expect that other energy components, such as dispersion and orbital terms, will be important players in the overall stabilization of the complexes studied herein (see the EDA and NBO analyses below).

### 2.2. Energetic Study

**Figure 2:**
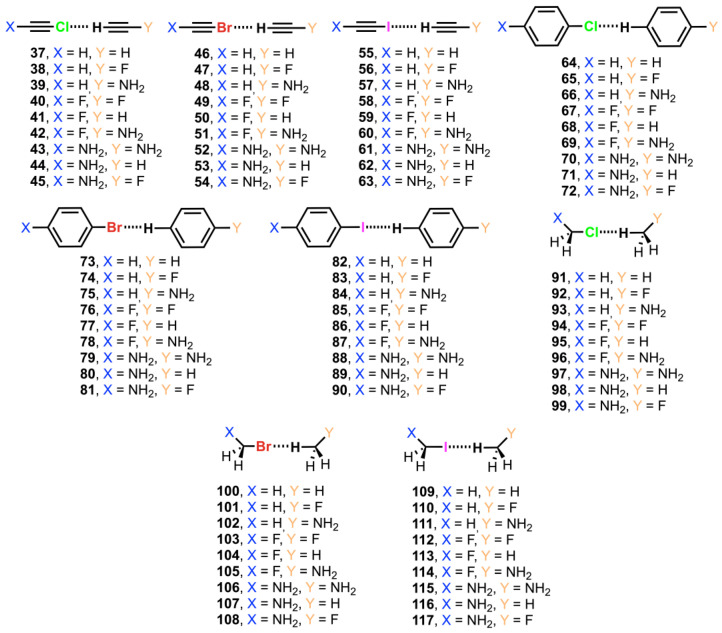
Complexes **37** to **117** used in this study.

**Figure 3:**
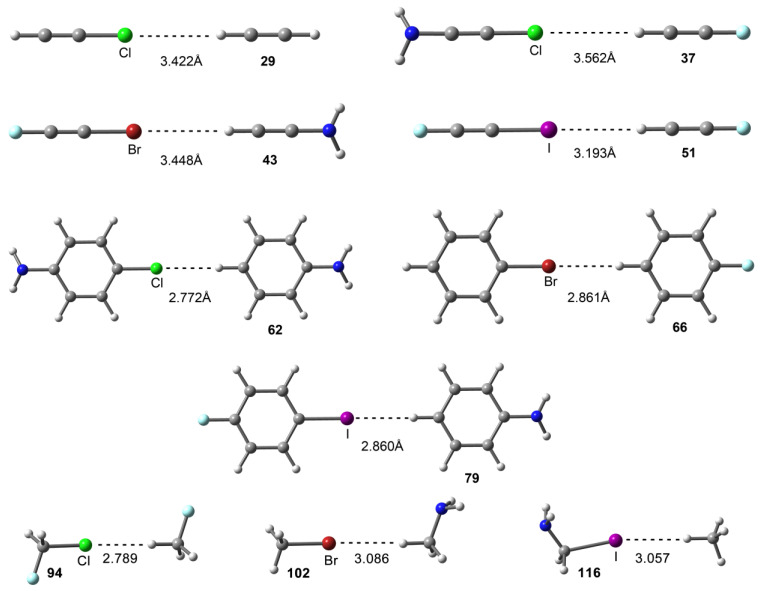
Optimized geometries of some representative complexes at the RI-MP2/aug-cc-pVQZ level of theory.

The interaction energies and equilibrium distances obtained for Complexes **37**–**117** are summarized in Table 2 (see also Figure 2 and Figure 3 as well as the Supplementary Information for the cartesian coordinates of all computed complexes). From the inspection of the results, some interesting points arose. Firstly, in most of the cases, the interaction energies were subtle but favorable (up to −9.5 kJ/mol). Secondly, a progressive strengthening of the interaction was generally observed ongoing from Cl- to I-involving complexes. This behavior was more clearly observed for sp and sp^2^ C atoms (Complexes **37** to **90**) than for sp^3^ C atoms (Complexes **91** to **117**). Lastly, substituent effects were almost negligible when using sp and sp^2^ C atom hybridizations (Complexes **37** to **90**), raising similar interaction energy values. On the contrary, we observed stronger variations in the interaction energy values when sp^3^ C atoms were used (Complexes **91** to **117**).

The most favorable complex among Cl sp carbon moieties was **45**, which involved –NH_2_ and –F groups and resulted in −0.83 kJ/mol. Complex **52** involving Br and two –NH_2_ groups achieved the most favorable interaction energy of this set (−0.55 kJ/mol). In addition, for both halogens, the interaction energy values were slightly repulsive when –F and –H substituents were included together in the same complex (Complexes **37**, **46**, **47**, **49**, and **50**), as expected from the MEP analysis discussed above, owing to an increase in the Hlg and H electrostatic potential values when these groups are present (see Table 1 above). Moreover, the presence of the –NH_2_ substituent favored complex formation for both the Cl and Br sets, either if it was attached to the Hlg- or H-bearing moiety (e.g., Complexes **39** and **42**–**45**), likely due to a lowering of the electrostatic repulsion between both electropositive regions (less positive electrostatic potential values). Lastly, for complexes involving iodine (**55** to **63**) the largest energy values involved –F and –NH_2_ substituents. In more detail, Complexes **60** and **61** obtained the most favorable interaction energy values of the I set (−3.01 and −2.97 kJ/mol, respectively), in line with the results obtained for their Cl and Br analogues. Owing to the very similar strength exhibited by these two complexes, we observed almost no influence of the electronic nature of the substituent attached to the halogen moiety on the stabilization of the complexes, since these two involved the same H-bearing moiety but a different halogenated moiety (substituted with either a –F or a –NH_2_ group). Finally, the rest of complexes belonging to this set (**55**, **56**, **58**, and **59**) also achieved favorable interaction energy values, but they were lower in magnitude compared to the –F/–NH_2_ combinations, indicating that the use of –F and –H substituents was less favored upon complexation, in line with the Cl and Br sets of complexes.

For carbon sp^2^ complexes (**64** to **90**), the interaction energy values obtained were attractive in all cases, ranging between −8.28 kJ/mol (Complex **90** involving I) and −3.66 kJ/mol (Complex **69** involving Cl). For Cl- and Br-involving complexes, similar interaction energy values were obtained, between −4.60 and −3.66 kJ/mol. In addition, for all three halogens considered, a subtle influence of substituent on the interaction energy strength was observed, in agreement with their carbon sp analogues. Lastly, for sp^3^ carbon complexes (**91** to **117**), the interaction energies obtained followed a reinforcement of the interaction from Cl/Br to I, in line with the noticeable changes observed in their respective MEP surface values (see Table 1 above). For instance, Complex **117** (involving I) achieved an interaction energy value of −9.5 kJ/mol, while its Cl and Br analogous (Complexes **99** and **108**) obtained interaction energy values of −3.9 and −3.5 kJ/mol, respectively. The only exceptions to this tendency were Complexes **111** (−0.9 kJ/mol) and **115** (−0.4 kJ/mol), which showed less favorable interaction energy values than their Cl and Br analogues. While the substituent effect was negligible in the Cl and Br series, we found that in the case of I it could remarkably affect the strength of the interaction, and those complexes involving –NH_2_ (**111**, **114**, and **115**) were disfavored compared to those involving –F and –H (e.g., Complexes **110** and **113**).

### 2.3. NBO, AIM, and NCIplot Analyses

To study if orbital contributions are important to explain the noncovalent complexes described above, we performed NBO calculations, focusing our attention on the second-order perturbation analysis, since it is a convenient tool to analyze donor–acceptor interactions. The results are summarized in Table 3 for a set of representative complexes.

Firstly, for sp C complexes (**43**, **45**, **58**, and **60**), the orbital contributions were of considerable strength compared to the total interaction energies, and depending on the halogen atom involved, a different number of orbital contributions were observed. For the two selected chlorine complexes (**43** and **45**) the orbital contributions were basically due to the interaction between bonding C-Cl orbitals (BD C-Cl) and empty Rydberg orbitals (Ry*) of carbon and hydrogen atoms belonging to the hydrogen-bearing moieties. On the other hand, for the selected bromine and iodine complexes (**53**, **54**, **58**, and **60**), a larger number of orbital interactions were observed. That is, in addition to the previously mentioned contribution, an interaction from a bonding C-Hlg orbital (BD C-Br/I) with an antibonding C-H orbital (BD*) was observed. Furthermore, orbital interactions between the bromine/iodine lone pair (LP) and antibonding C-H orbitals (BD*) were also observed, being the most remarkable contribution in these complexes. Finally, for iodine complexes (**58** and **60**), a back bonding donating effect was observed from the bonding C-H orbitals (BD C-H) to the antibonding C-I orbitals (BD* C-I). These results agree with those retrieved from the energetic study discussed above, which showed larger binding energy values for the iodine set over bromine- and chlorine-involving complexes.

Among the selected sp^2^ C complexes (**65**, **70**, **77**, and **81**), the orbital contributions were also noticeable compared to the total interaction energies and they were supported by the interaction between a halogen lone pair (LP) with an antibonding C-H orbital (BD* C-H). In addition, an orbital contribution from the bonding C-Hlg orbitals (BD Cl/Br/I) to the antibonding C-H orbitals (BD* C-H) was also observed, similar to the sp C examples. Finally, a back bonding donating effect was also observed between the bonding C-H orbitals (BD C-H) and the antibonding C-Hlg orbitals (BD* Cl/Br/I).

Lastly, among the sp^3^ C complexes (**92**, **95**, **103**, and **110**), the orbital contributions were of remarkable magnitude compared to the interaction energies retrieved and were based on the interaction between the bonding C-Hlg orbitals (BD Cl/Br) and the antibonding C-H orbitals (BD* C-H) from the H-bearing molecule. In addition, the halogen lone pairs (LP Cl/Br/I) also contributed to the overall orbital stabilization by interacting with the antibonding C-H orbitals (BD* C-H). Finally, no back bonding donation effects involving the antibonding C-Hlg orbital were observed involving sp^3^ C complexes.

**Figure 4:**
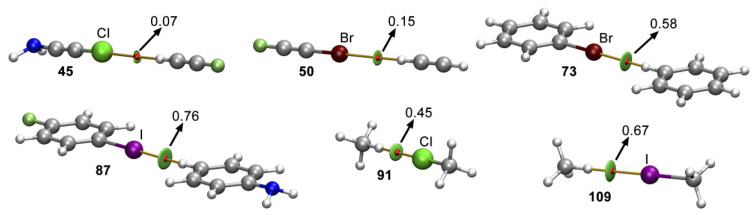
Distribution of intermolecular bond critical points (red spheres) and bond paths in Complexes **45**, **50**, **73**, **87**, **91**, and **109**. The values of the density (ρ·10^2^) related to the C-Hlg···H-C interaction are also included in a.u. NCIPlot color range −0.02 au ≤ (sign λ_2_)ρ ≤ +0.02 a.u.

We have also used the QTAIM methodology in conjunction with the NCIplot technique to characterize the nonbonded complexes described above, and the results of some representative cases are shown in Figure 4. As it can be observed, in all cases, the C-Hlg···H-C interaction was characterized by the presence of a bond path and a bond critical point (BCP) that connected both halogen and hydrogen atoms, along with a small green isosurface, which was useful to assess the weak nature of the interactions described herein, as well as their extension in real space. Lastly, the values of the Laplacian in all cases were positive, as is common in closed-shell calculations.

**Figure 5:**
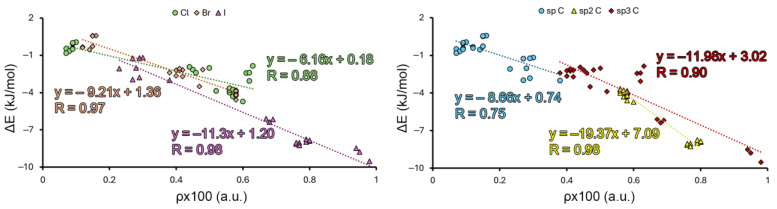
Graphical representations of the values of electron density at the BCP that characterize the Hlg···H interaction (ρx100 in a.u.) vs. the interaction energy values (ΔE in kJ/mol) for Complexes **37** to **117**.

In Figure 5, we have presented the values of electron density at the BCP that characterizes the Hlg···H interaction (ρx100 in a.u.) vs. the interaction energy values (ΔE in kJ/mol) for Complexes **37** to **117**. As noticed, in all the cases, we obtained very good correlation coefficients (R = 0.88, 0.97, and 0.98 for Cl, Br, and I, respectively), which demonstrated the direct relationship between the strength of the Hlg···H interaction and their corresponding BCP density value. In the case of the I series, we discarded Complexes **111**, **114**, and **115**, since their ρx100 values completely distorted the correlation.

If the same representation was carried out by considering each C hybridization, we obtained a modest correlation coefficient in the case of the sp C hybridization (R = 0.75), while for sp^2^ and sp^3^ C hybridizations, we obtained correlation coefficient values of 0.98 and 0.90, respectively. It is also worth noting that in the case of the sp^2^ C hybridization, the data values were clustered in two regions (yellow triangles in the right graph). Therefore, more points between these two clusters should be incorporated in this case to be fully sure of this result.

### 2.4. EDA Analysis

To further investigate physical nature of the C-Hlg···H-C interaction energies obtained, we performed an EDA analysis on several representative examples (Complexes **46**, **64**, **73**, **82**, and **100** involving sp, sp^2^, and sp^3^ Br derivatives and sp^2^ Cl and I moieties). Complexes **47**, **73**, and **100** allowed us to compare the effect of the C hybridization, since in all these three complexes, the Hlg used was Br. On the other hand, Complexes **64** and **82** corresponded both to a sp^2^ C atom using either Cl or I as the halogen, allowing us to compare the effect of having a light or a heavy halogen in the structure.

**Figure 6:**
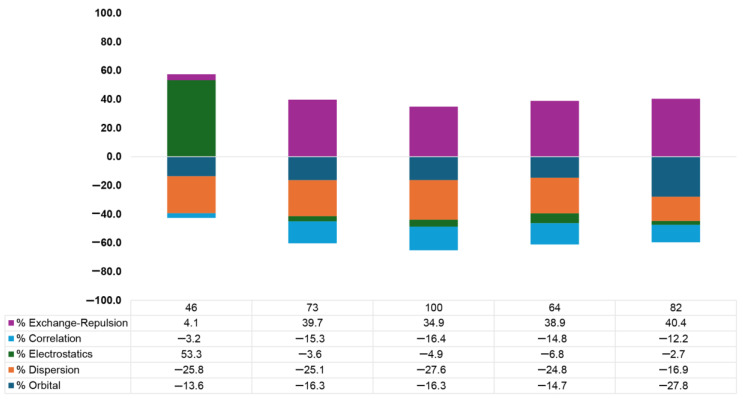
Energy Decomposition Analysis of the exchange–repulsion, correlation, electrostatic, dispersion and orbital terms in percentages for Complexes **46**, **73**, **100**, **64**, and **82**.

The energy partition scheme used unveiled the contributions of the electrostatic, exchange–repulsion, orbital, dispersion, and electron correlation terms as percentages (see Figure 6). As noted, for Complexes **73**, **64**, **82**, and **100**, the electrostatic term was slightly favorable, while for Complex **46**, it showed a positive value, likely due to the more positive σ-hole MEP values exhibited by the sp C halogenated derivatives (see Table 1 above). On the other hand, dispersion and orbital contributions were the main stabilizing factors among these complexes, in line with what was observed from the NBO analysis below. Lastly, the correlation contribution was also favorable in all these five selected complexes, although it was of lower magnitude than dispersion and orbital terms.

A comparison of complexes containing the same halogen (complexes **46**, **73**, and **100**) pointed to the role of electrostatics and electron correlation as the more variable factors, since the dispersion and orbital terms were of similar magnitude among the three complexes considered. On the other hand, changing both the halogen and type of hybridization (Complexes **64**, **82**, and **100**) strongly affected the dispersion and orbital contributions, exhibiting opposite tendencies between these three selected complexes. 

## 3. Materials and Methods

The energies of all complexes included in this study were computed at the RI-MP2 [34,35]/aug-cc-pVQZ [36] level of theory. For the iodine atom, the aug-cc-pVQZ-PP basis set was used. The calculations were performed using the program TURBOMOLE version 7.7 [37]. After optimization of the monomers and supramolecular complexes, the interaction energy values gathered in Table 2 were obtained using the following supramolecular approximation: ∆E = E_complex_ − E_monomer1_ − E_monomer2_. The Cs and C2v symmetry point groups were imposed during the optimization process. The use of symmetry allowed us to keep the Hlg/H atom σ-holes one in front of the other, which was the main topic of this study. Otherwise, the main preferred conformation would be likely based on the interaction between the Hlg negative belt and the electropositive H atom (a classical hydrogen bond). All the structures presented herein correspond to converged geometries. Frequency calculations were performed at the RI-MP2/aug-cc-pVQZ level of theory.

The MEP (molecular electrostatic potential) surfaces were performed at the MP2/aug-cc-pVQZ level of theory by means of the Gaussian 16 calculation package [38] and analyzed using the Gaussview 5.0 program [39]. The calculations for the wavefunction analysis were also carried out at the MP2/aug-cc-pVQZ level of theory (also using the Gaussian 16 software) and analyzed by means of the AIMall software Version 19.10.12 [40]. The NBO [41] analyses were performed at the HF/def2-TZVP level of theory using the NBO 7.0 program [42]. In addition, the Energy Decomposition Analysis (EDA) [43,44] scheme was used to understand the roles of the electrostatic, exchange–repulsion, orbital, dispersion, and electron correlation contributions in the formation of the noncovalent complexes studied herein at the PBE0-D3/aug-cc-pVQZ level of theory, also using TURBOMOLE 7.7 software. Lastly, the NCIplot [45] isosurfaces correspond to both favorable and unfavorable interactions, as differentiated by the sign of the second-density Hessian eigenvalue and defined by the isosurface color. The color scheme is a red−yellow−green−blue scale, with red for repulsive (ρ_cut_^+^) and blue for attractive (ρ_cut_^−^) NCI interaction densities. Yellow and green surfaces correspond to weak repulsive and weak attractive interactions, respectively. The surfaces were visualized using Visual Molecular Dynamics (VMD) software Version 1.9.4 [46]. All these calculations (MEP, NBO, EDA, QTAIM, and NCIplot) were performed using the optimized geometries at the RI-MP2/aug-cc-pVQZ level of theory taken from the TURBOMOLE software Version 7.7.

## 4. Conclusions

In this work, C-Hlg···H-C interactions encompassing C-Hlg (Hlg = Cl, Br, and I) and H-C moieties that involved different C hybridizations were studied, revealing their subtle but attractive nature, with interaction energies up to −9.5 kJ/mol. In more detail, complexes involving sp hybridized C atoms obtained the lowest interaction energy values of the study (e.g., −0.83 kJ/mol for Complex **45** and −0.55 kJ/mol for Complex **52**). This was followed by sp^2^ hybridized C atoms (e.g., −8.28 kJ/mol for Complex **90** and −3.66 kJ/mol for Complex **69**) and, lastly, sp^3^ hybridized C atoms (e.g., −9.5 kJ/mol for Complex **117** and −3.90 kJ/mol for Complex **99**). The results also showed a progressive reinforcement of the interaction strength ongoing from chlorine- to iodine-involving complexes, likely due to the larger polarizability value exhibited by heavier halogens that counterbalanced the electrostatic repulsion between both interacting moieties. Additionally, the influences of the –F, –H, and –NH_2_ substituents on the interaction energy values were almost negligible in most cases, achieving similar interaction energy values for all combinations involving a particular halogenated moiety. Lastly, the EDA and NBO analyses showed that dispersion and orbital contributions played important roles in the overall stabilization of the complexes, while the QTAIM and NCIplot techniques allowed the visualization of the charge–density properties of these very weak noncovalent bonds. We believe that our study might be of importance for those chemists working in the supramolecular chemistry, crystal engineering, or rational drug design fields. Although the main point of this study was to understand the physical nature of these very weak noncovalent Hlg···H contacts from a theoretical perspective, an analysis of the Cambridge Structural Database (CSD) is currently ongoing in our group to find experimental evidence of the existence of such interactions in solid-state chemistry.

## Figures and Tables

**Table 1:** Electrostatic potential values measured at the Hlg/H atom for Compounds **1** to **36** in kJ/mol.

	–Cl	–Br	–I	–H
**sp C**	**1** (X = H) = +87.7 **2** (X = F) = +92.0 **3** (X = NH_2_) = +61.4	**4** (X = H) = +112.2 **5** (X = F) = +116.2 **6** (X = NH_2_) = +86.0	**7** (X = H) = +138.0 **8** (X = F) = +144.4 **9** (X = NH_2_) = +108.7	**10** (X = H) = +138.3 **11** (X = F) = +148.3 **12** (X = NH_2_) = +106.4
**sp^2^ C**	**13** (X = H) = +20.0 **14** (X = F) = +31.5 **15** (X = NH_2_) = +9.0	**16** (X = H) = +41.3 **17** (X = F) = +53.1 **18** (X = NH_2_) = +30.5	**19** (X = H) = +64.3 **20** (X = F) = +76.4 **21** (X = NH_2_) = +50.2	**22** (X = H) = +63.6 **23** (X = F) = +78.0 **24** (X = NH_2_) = +51.4
**sp^3^ C**	**25** (X = H) = −0.5 **26** (X = F) = +30.2 **27** (X = NH_2_) = −21.6	**28** (X = H) = +24.3 **29** (X = F) = +49.8 **30** (X = NH_2_) = −8.9	**31** (X = H) = +57.0 **32** (X = F) = +71.1 **33** (X = NH_2_) = −7.3	**34** (X = H) = +38.4 **35** (X = F) = +77.8 **36** (X = NH_2_) = +39.7

**Table 2:** Interaction energies of Complexes **37** to **117** at the RI-MP2/aug-cc-pVQZ level of theory (ΔE in kJ/mol), equilibrium distances (Re in Å), and value of the density at the bond critical point (ρ·10^2^, a.u.) that characterizes the Hlg···H interaction. The symmetry point group used during the optimization process (Symm.) and the number of imaginary frequencies obtained (N_imag_) are also indicated.

Complex	ΔE	R_e_	ρ·10^2^	Symm.	N_imag_
**37**	+0.06	3.422	0.10	Cs	0
**38**	−3·10^−2^	3.445	0.09	Cs	0
**39**	−0.44	3.452	0.09	Cs	0
**40**	−0.06	3.461	0.09	Cs	0
**41**	−0.01	3.439	0.09	Cs	0
**42**	−0.48	3.560	0.07	Cs	0
**43**	−0.68	3.508	0.08	Cs	0
**44**	−0.51	3.471	0.09	Cs	0
**45**	−0.83	3.562	0.07	Cs	0
**46**	+0.58	3.308	0.16	Cs	0
**47**	+0.56	3.331	0.15	Cs	0
**48**	−0.28	3.337	0.15	Cs	0
**49**	+0.53	3.347	0.15	Cs	0
**50**	+0.54	3.326	0.15	Cs	0
**51**	−0.33	3.448	0.12	Cs	0
**52**	−0.55	3.384	0.14	Cs	0
**53**	−0.28	3.357	0.15	Cs	0
**54**	−0.40	3.440	0.12	Cs	0
**55**	−1.19	3.176	0.30	Cs	1
**56**	−1.22	3.196	0.29	Cs	1
**57**	−2.81	3.202	0.29	Cs	1
**58**	−1.26	3.125	0.27	Cs	1
**59**	−1.24	3.194	0.29	Cs	1
**60**	−3.01	3.074	0.38	Cs	2
**61**	−2.97	3.234	0.27	Cs	1
**62**	−2.03	3.216	0.28	Cs	1
**63**	−2.09	3.294	0.23	Cs	1
**64**	−3.98	2.771	0.57	C2v	0
**65**	−4.31	2.768	0.57	C2v	0
**66**	−3.69	2.780	0.56	Cs	0
**67**	−4.00	2.773	0.56	C2v	0
**68**	−3.81	2.776	0.56	C2v	0
**69**	−3.66	2.781	0.56	Cs	0
**70**	−3.77	2.772	0.57	Cs	0
**71**	−4.22	2.762	0.58	Cs	0
**72**	−4.72	2.751	0.60	Cs	0
**73**	−3.99	2.860	0.58	Cs	0
**74**	−4.23	2.861	0.58	C2v	0
**75**	−3.83	2.861	0.58	Cs	0
**76**	−3.94	2.861	0.58	C2v	0
**77**	−3.85	2.861	0.58	C2v	0
**78**	−3.83	2.861	0.58	Cs	0
**79**	−3.88	2.862	0.58	Cs	0
**80**	−4.21	2.860	0.58	Cs	0
**81**	−4.60	2.860	0.58	Cs	0
**82**	−7.91	2.839	0.80	C2v	1
**83**	−7.98	2.841	0.79	C2v	1
**84**	−8.07	2.860	0.76	Cs	1
**85**	−7.76	2.839	0.79	C2v	1
**86**	−7.85	2.834	0.80	C2v	1
**87**	−8.15	2.860	0.76	Cs	1
**88**	−8.04	2.860	0.76	Cs	1
**89**	−8.05	2.856	0.77	Cs	1
**90**	−8.28	2.858	0.77	Cs	1
**91**	−2.16	2.933	0.45	Cs	0
**92**	−3.07	2.783	0.62	Cs	0
**93**	−1.85	2.788	0.63	Cs	1
**94**	−2.44	2.789	0.61	Cs	0
**95**	−1.94	2.942	0.44	Cs	0
**96**	−2.40	2.795	0.62	Cs	1
**97**	−2.03	2.928	0.50	Cs	1
**98**	−2.40	2.959	0.46	Cs	0
**99**	−3.88	2.901	0.52	Cs	0
**100**	−2.22	3.089	0.40	Cs	0
**101**	−2.65	3.087	0.40	Cs	0
**102**	−2.18	3.086	0.42	Cs	1
**103**	−2.25	3.088	0.41	Cs	0
**104**	−2.09	3.091	0.41	Cs	0
**105**	−2.71	3.089	0.42	Cs	1
**106**	−2.15	3.070	0.48	Cs	0
**107**	−2.41	3.165	0.38	Cs	0
**108**	−3.51	3.071	0.47	Cs	0
**109**	−6.11	3.008	0.67	Cs	0
**110**	−8.78	2.849	0.95	Cs	0
**111**	−0.92	2.738	1.20	Cs	0
**112**	−8.50	2.860	0.94	Cs	0
**113**	−6.18	3.003	0.69	Cs	0
**114**	−2.34	2.728	1.23	Cs	0
**115**	−0.44	2.790	1.15	Cs	0
**116**	−6.38	3.057	0.68	Cs	0
**117**	−9.53	2.869	0.98	Cs	1

**Table 3:** Donor and acceptor NBOs with indications of the second-order interaction energy E^(2)^ in kJ/mol and type of interaction for some representative complexes.

Complex	Donor ^1^	Acceptor ^1^	E^2^	Type
**43**	BD C-Cl BD C-Cl	Ry* C Ry* H	0.46 0.71	σ→Ry* σ→Ry*
**45**	BD C-Cl BD C-Cl	Ry* C Ry* H	0.33 0.50	σ→Ry* σ→Ry*
**53**	BD C-Br LP Br	BD* C-H BD* C-H	0.33 0.29	σ→σ* n→σ*
**54**	BD C-Br LP Br	BD* C-H BD* C-H	0.29 0.33	σ→σ* n→σ*
**58**	BD C-I LP I BD C-H	BD* C-H BD* C-H BD* C-I	0.54 1.76 0.25	σ→σ* n→σ* σ→σ*
**60**	BD C-I LP I BD C-H	BD* C-H BD* C-H BD* C-I	0.67 3.09 0.50	σ→σ* n→σ* σ→σ*
**65**	BD C-Cl LP Cl BD C-H	BD* C-H BD* C-H BD* C-Cl	0.75 4.51 0.54	σ→σ* n→σ* σ→σ*
**70**	BD C-Cl LP Cl BD C-H	BD* C-H BD* C-H BD* C-Cl	0.59 4.31 0.59	σ→σ* n→σ* σ→σ*
**77**	BD C-Br LP Br BD C-H	BD* C-H BD* C-H BD* C-Br	1.25 4.10 0.96	σ→σ* n→σ* σ→σ*
**81**	BD C-Br LP Br BD C-H	BD* C-H BD* C-H BD* C-Br	1.30 4.26 0.88	σ→σ* n→σ* σ→σ*
**87**	BD C-I LP I BD C-H	BD* C-H BD* C-H BD* C-I	1.92 6.65 2.59	σ→σ* n→σ* σ→σ*
**89**	BD C-I LP I BD C-H	BD* C-H BD* C-H BD* C-I	2.10 6.86 2.38	σ→σ* n→σ* σ→σ*
**92**	LP Cl BD C-Cl	BD* C-H Ry* H	1.19 1.03	n→σ* σ→Ry*
**95**	BD C-Cl LP Cl	Ry* H BD* C-H	1.01 0.36	σ→Ry* n→σ*
**103**	BD C-Br LP Br	Ry* H BD* C-H	0.81 0.51	σ→Ry* n→σ*
**110**	LP I	BD* C-H	2.44	n→σ*

^1^ BD, BD*, LP, and Ry* stand for bonding, anti-bonding, lone pair, and empty Rydberg orbital, respectively.

## Data Availability

The data needed to reproduce the results derived from this study can be found in the electronic Supporting Information.

## Supplementary Materials

The following supporting information can be downloaded at https://www.mdpi.com/article/10.3390/molecules29235606/s1: Cartesian coordinates of Complexes **37** to **117**.
