# Supplementary material for: Unconventional C—Hlg···H–C (Hlg = Cl, Br, and I) Interactions Involving Organic Halides: A Theoretical Study
Source: Molecules. 2024 Nov 27;29(23):5606. doi: 10.3390/molecules29235606 (PMC11643732; doi:10.3390/molecules29235606)
Supplement: Supplementary file 1 [file molecules-29-05606-s001.zip › molecules-3318597-supplementary.pdf]

Unconventional C–Hlg···H–C (Hlg = Cl, Br and I) Interactions Involving Organic Halides: A Theoretical Study

Sergi Burguera and Antonio Bauzá *

Department of Chemistry, Universitat de les Illes Balears, Ctra. de Valldemossa km 7.5, 07122 Palma, Baleares, Spain

* Correspondence: antonio.bauza@uib.es

Electronic Supplementary Information

Cartesian coordinates of complexes **37** to **117**

37.

C	-4.3401295	0.0008868	0.0000000
C	-3.1296832	-0.0002945	0.0000000
Cl	-1.4960987	-0.0018172	0.0000000
C	2.9870197	0.0001096	0.0000000
H	1.9260774	0.0016205	0.0000000
C	4.1961543	-0.0006808	0.0000000
H	5.2575281	-0.0014202	0.0000000
H	-5.4008682	0.0015957	0.0000000

38.

C	-4.3758697	0.0007989	0.0000000
C	-3.1654334	-0.0002577	0.0000000
Cl	-1.5317997	-0.0017670	0.0000000
C	2.9715517	0.0004406	0.0000000
H	1.9130420	0.0014171	0.0000000
C	4.1739710	-0.0006098	0.0000000
H	-5.4365920	0.0017034	0.0000000
F	5.4511300	-0.0017255	0.0000000

39.

C	-5.5791147	-0.0492154	0.0000000
C	-4.3685743	-0.0598785	0.0000000
Cl	-2.7351009	-0.0738023	0.0000000
C	1.7745341	-0.0993976	0.0000000
H	0.7159492	-0.1335121	0.0000000
C	2.9846690	-0.0579372	0.0000000
N	4.3397903	-0.0898525	0.0000000
H	4.7538058	0.3018312	-0.8311474

H	4.7538058	0.3018312	0.8311474
H	-6.6397643	-0.0400667	0.0000000

40.

C	-4.3520890	0.0007601	0.0000000
C	-3.1505924	-0.0002622	0.0000000
Cl	-1.5132554	-0.0017256	0.0000000
C	3.0058828	0.0004045	0.0000000
H	1.9473477	0.0013363	0.0000000
C	4.2083168	-0.0006388	0.0000000
F	-5.6310679	0.0018678	0.0000000
F	5.4854575	-0.0017422	0.0000000

41.

C	-4.3170590	0.0007298	0.0000000
C	-3.1155584	-0.0002909	0.0000000
Cl	-1.4782653	-0.0017597	0.0000000
C	3.0220608	0.0003870	0.0000000
H	1.9610902	0.0013278	0.0000000
C	4.2312127	-0.0006612	0.0000000
H	5.2926406	-0.0015723	0.0000000
F	-5.5961216	0.0018393	0.0000000

42.

C	-4.4163811	-0.0669600	0.0000000
Cl	-2.7790266	-0.0784299	0.0000000
C	1.8392705	-0.0962858	0.0000000
H	0.7805671	-0.1321024	0.0000000
C	3.0493850	-0.0535483	0.0000000
C	-5.6179171	-0.0584778	0.0000000
F	-6.8973819	-0.0493735	0.0000000
N	4.4045206	-0.0830418	0.0000000
H	4.8184818	0.3091097	-0.8309424
H	4.8184818	0.3091097	0.8309424

43.

C	-4.3596859	0.0837346	0.0000000
C	-3.1503032	0.1299774	0.0000000
Cl	-1.5116463	0.1785590	0.0000000
C	3.0548955	0.1512402	0.0000000
H	1.9962315	0.1929286	0.0000000
C	4.2647220	0.1017906	0.0000000
N	-5.7159465	0.1223879	0.0000000
H	-6.1304609	-0.2711248	-0.8304897
H	-6.1304609	-0.2711248	0.8304897
N	5.6205168	0.1245387	0.0000000
H	6.0310690	-0.2714536	0.8308717
H	6.0310690	-0.2714536	-0.8308717

44.

C	-3.1124849	0.0248600	0.0000000
C	-1.9000122	0.0658553	0.0000000
Cl	-0.2615518	0.1193740	0.0000000
C	4.2732547	0.0983556	0.0000000
H	3.2090192	0.0981823	0.0000000
C	5.4854910	0.0984704	0.0000000
N	-4.4703376	0.0620679	0.0000000
H	-4.8866933	-0.3330066	-0.8339285
H	-4.8866933	-0.3330066	0.8339285
H	6.5500081	0.0988477	0.0000000

45.

C	-1.9560033	0.0827738	0.0000000
Cl	-0.3172401	0.1311666	0.0000000
C	4.3029333	0.0722978	0.0000000
H	3.2440416	0.0786158	0.0000000
C	5.5053800	0.0648776	0.0000000
C	-3.1655140	0.0411563	0.0000000
N	-4.5210312	0.0843262	0.0000000
H	-4.9377889	-0.3060974	-0.8308116
H	-4.9377889	-0.3060974	0.8308116
F	6.7830115	0.0569807	0.0000000

46.

C	-4.3800509	0.0008013	0.0000000
C	-3.1679288	-0.0002427	0.0000000
Br	-1.3810506	-0.0018831	0.0000000
C	2.9878370	0.0004160	0.0000000
H	1.9272781	0.0013902	0.0000000
C	4.1968881	-0.0006355	0.0000000
H	5.2583158	-0.0015469	0.0000000
H	-5.4412886	0.0017006	0.0000000

47.

C	-4.4155927	0.0008378	0.0000000
C	-3.2034382	-0.0002103	0.0000000
Br	-1.4164515	-0.0019025	0.0000000
C	2.9722225	0.0004320	0.0000000
H	1.9141268	0.0014423	0.0000000
C	4.1745850	-0.0006078	0.0000000
H	-5.4768792	0.0017373	0.0000000
F	5.4514273	-0.0017287	0.0000000

48.

C	-5.6187907	-0.0488832	0.0000000
C	-4.4065305	-0.0596808	0.0000000
Br	-2.6193984	-0.0747200	0.0000000
C	1.7749320	-0.0992038	0.0000000
H	0.7167480	-0.1338159	0.0000000

C	2.9850418	-0.0577815	0.0000000
N	4.3398366	-0.0894521	0.0000000
H	4.7540819	0.3016006	-0.8313217
H	4.7540819	0.3016006	0.8313217
H	-6.6800026	-0.0396639	0.0000000

49.

C	-4.3922132	0.0007989	0.0000000
C	-3.1883702	-0.0002229	0.0000000
Br	-1.3989913	-0.0018522	0.0000000
C	3.0065545	0.0003963	0.0000000
H	1.9484791	0.0013634	0.0000000
C	4.2089345	-0.0006373	0.0000000
F	-5.6701828	0.0019003	0.0000000
F	5.4857893	-0.0017465	0.0000000

50.

C	-4.3572253	0.0007688	0.0000000
C	-3.1533700	-0.0002509	0.0000000
Br	-1.3639357	-0.0018792	0.0000000
C	3.0227056	0.0003800	0.0000000
H	1.9621619	0.0013500	0.0000000
C	4.2317711	-0.0006635	0.0000000
H	5.2931749	-0.0015737	0.0000000
F	-5.6352825	0.0018686	0.0000000

51.

C	-4.4553605	-0.0667639	0.0000000
Br	-2.6656074	-0.0790095	0.0000000
C	1.8405089	-0.0962612	0.0000000
H	0.7822070	-0.1329159	0.0000000
C	3.0505697	-0.0532850	0.0000000
C	-5.6593109	-0.0581747	0.0000000
F	-6.9377727	-0.0490666	0.0000000
N	4.4054441	-0.0824272	0.0000000
H	4.8196609	0.3089520	-0.8311371
H	4.8196609	0.3089520	0.8311371

52.

C	-4.3876658	0.0837971	0.0000000
C	-3.1761430	0.1298289	0.0000000
Br	-1.3858198	0.1834110	0.0000000
C	3.0564059	0.1509070	0.0000000
H	1.9981856	0.1937457	0.0000000
C	4.2662595	0.1013767	0.0000000
N	-5.7424802	0.1192753	0.0000000
H	-6.1577265	-0.2718887	-0.8313593
H	-6.1577265	-0.2718887	0.8313593
N	5.6218664	0.1241190	0.0000000
H	6.0324222	-0.2713416	0.8311289

H	6.0324222	-0.2713416	-0.8311289

53.

C	-3.1408412	0.0203926	0.0000000
C	-1.9293198	0.0641498	0.0000000
Br	-0.1397171	0.1282158	0.0000000
C	4.2775819	0.0980837	0.0000000
H	3.2165995	0.0968775	0.0000000
C	5.4867528	0.0986049	0.0000000
N	-4.4949895	0.0570571	0.0000000
H	-4.9121141	-0.3312117	-0.8316465
H	-4.9121141	-0.3312117	0.8316465
H	6.5481614	0.0990420	0.0000000

54.

C	-1.9819656	0.0798665	0.0000000
Br	-0.1919818	0.1385910	0.0000000
C	4.3064029	0.0722886	0.0000000
H	3.2478323	0.0777284	0.0000000
C	5.5087667	0.0648623	0.0000000
C	-3.1937402	0.0401587	0.0000000
N	-4.5477796	0.0808167	0.0000000
H	-4.9668452	-0.3056987	-0.8315126
H	-4.9668452	-0.3056987	0.8315126
F	6.7861557	0.0570852	0.0000000

55.

C	-4.4266542	0.0008348	0.0000000
C	-3.2125852	-0.0001559	0.0000000
I	-1.2475942	-0.0021054	0.0000000
C	2.9888440	0.0004275	0.0000000
H	1.9287980	0.0014568	0.0000000
C	4.1978976	-0.0006507	0.0000000
H	5.2593733	-0.0015547	0.0000000
H	-5.4880794	0.0017477	0.0000000

56.

C	-4.4605335	0.0008550	0.0000000
C	-3.2464777	-0.0001487	0.0000000
I	-1.2815124	-0.0019851	0.0000000
C	2.9717292	0.0004263	0.0000000
H	1.9142183	0.0014077	0.0000000
C	4.1740809	-0.0006115	0.0000000
H	-5.5219670	0.0017702	0.0000000
F	5.4504623	-0.0017140	0.0000000

57.

C	-5.6640310	-0.0480103	-0.0000038
C	-4.4498623	-0.0597401	0.0000051

I	-2.4841802	-0.0763434	-0.0000000
C	1.7747439	-0.0991692	-0.0000037
H	0.7170264	-0.1333979	-0.0000003
C	2.9848635	-0.0574469	0.0000046
N	4.3391161	-0.0887412	-0.0000008
H	4.7538410	0.3010513	-0.8316542
H	4.7538437	0.3010512	0.8316536
H	-6.7253610	-0.0392535	-0.0000004

58.

C	-4.4379738	0.0008176	0.0000000
C	-3.2313138	-0.0001566	0.0000000
I	-1.2655924	-0.0019926	0.0000000
C	3.0065006	0.0003960	0.0000000
H	1.9489816	0.0013724	0.0000000
C	4.2088657	-0.0006398	0.0000000
F	-5.7147361	0.0019463	0.0000000
F	5.4852682	-0.0017433	0.0000000

59.

C	-4.4032651	0.0007868	0.0000000
C	-3.1966037	-0.0001773	0.0000000
I	-1.2308272	-0.0020441	0.0000000
C	3.0228067	0.0003884	0.0000000
H	1.9627339	0.0013749	0.0000000
C	4.2318688	-0.0006724	0.0000000
H	5.2933815	-0.0015743	0.0000000
F	-5.6800949	0.0019180	0.0000000

60.

C	-4.3539482	-0.0660331	0.0000000
I	-2.3875713	-0.0705772	0.0000000
C	1.7438498	-0.0898707	0.0000000
H	0.6864023	-0.1180300	0.0000000
C	2.9540618	-0.0526752	0.0000000
C	-5.5607194	-0.0596841	0.0000000
F	-6.8380623	-0.0547622	0.0000000
N	4.3079917	-0.0884629	0.0000000
H	4.7239979	0.3000477	-0.8315936
H	4.7239979	0.3000477	0.8315936

61.

C	-4.4158491	0.0797772	0.0000000
C	-3.2018210	0.1204324	0.0000000
I	-1.2368971	0.1959225	0.0000000
C	3.0549625	0.1533166	0.0000000
H	1.9968945	0.1906040	0.0000000
C	4.2647190	0.1024384	0.0000000
N	-5.7685662	0.1157560	0.0000000
H	-6.1873951	-0.2693231	-0.8320712

H	-6.1873951	-0.2693231	0.8320712
N	5.6198914	0.1234195	0.0000000
H	6.0307282	-0.2715102	0.8312173
H	6.0307282	-0.2715102	-0.8312173

62.

C	-3.1731854	0.0173204	0.0000000
C	-1.9592635	0.0564209	0.0000000
I	0.0045237	0.1395695	0.0000000
C	4.2810292	0.0980200	0.0000000
H	3.2204394	0.0957984	0.0000000
C	5.4902119	0.0989889	0.0000000
N	-4.5251602	0.0535543	0.0000000
H	-4.9451713	-0.3293693	-0.8324919
H	-4.9451713	-0.3293693	0.8324919
H	6.5517474	0.0990662	0.0000000

63.

C	-2.0096065	0.0713601	0.0000000
I	-0.0455236	0.1501646	0.0000000
C	4.3060488	0.0721846	0.0000000
H	3.2478301	0.0761881	0.0000000
C	5.5084900	0.0650583	0.0000000
C	-3.2236653	0.0361748	0.0000000
N	-4.5754216	0.0777751	0.0000000
H	-4.9968368	-0.3030903	-0.8327055
H	-4.9968368	-0.3030903	0.8327055
F	6.7855217	0.0572749	0.0000000

64.

C	0.0000000	0.0000000	5.5955701
C	1.2038918	0.0000000	4.8978318
C	1.2122764	0.0000000	3.5068364
C	0.0000000	0.0000000	2.8274510
C	-1.2122764	0.0000000	3.5068364
C	-1.2038918	0.0000000	4.8978318
H	2.1426479	0.0000000	5.4338617
H	2.1391579	0.0000000	2.9520105
H	-2.1391579	0.0000000	2.9520105
H	-2.1426479	0.0000000	5.4338617
C	0.0000000	0.0000000	-2.7566671
C	1.2055057	0.0000000	-3.4520729
C	1.2055163	0.0000000	-4.8439539
C	0.0000000	0.0000000	-5.5398197
C	-1.2055163	0.0000000	-4.8439539
C	-1.2055057	0.0000000	-3.4520729
H	0.0000000	0.0000000	-1.6759154
H	2.1412393	0.0000000	-2.9102694
H	2.1418022	0.0000000	-5.3848069
H	-2.1418022	0.0000000	-5.3848069
H	-2.1412393	0.0000000	-2.9102694
H	0.0000000	0.0000000	-6.6210929

H	0.0000000	0.0000000	6.6761301
Cl	0.0000000	0.0000000	1.0954691

65.

C	0.0000000	0.0000000	5.6047357
C	1.2039176	0.0000000	4.9070586
C	1.2123946	0.0000000	3.5160549
C	0.0000000	0.0000000	2.8370353
C	-1.2123946	0.0000000	3.5160549
C	-1.2039176	0.0000000	4.9070586
H	2.1426529	0.0000000	5.4430902
H	2.1393035	0.0000000	2.9613114
H	-2.1393035	0.0000000	2.9613114
H	-2.1426529	0.0000000	5.4430902
C	0.0000000	0.0000000	-2.7432068
C	1.2045988	0.0000000	-3.4397338
C	1.2133672	0.0000000	-4.8312461
C	0.0000000	0.0000000	-5.4966863
C	-1.2133672	0.0000000	-4.8312461
C	-1.2045988	0.0000000	-3.4397338
H	0.0000000	0.0000000	-1.6633472
H	2.1419008	0.0000000	-2.9015144
H	2.1332422	0.0000000	-5.3971829
H	-2.1332422	0.0000000	-5.3971829
H	-2.1419008	0.0000000	-2.9015144
F	0.0000000	0.0000000	-6.8440879
H	0.0000000	0.0000000	6.6852886
Cl	0.0000000	0.0000000	1.1045927

66.

C	-6.1852515	0.0317118	0.0000000
C	-5.4874207	0.0297547	1.2038504
C	-4.0964425	0.0259611	1.2121645
C	-3.4166372	0.0241182	0.0000000
C	-4.0964425	0.0259611	-1.2121645
C	-5.4874207	0.0297547	-1.2038504
H	-6.0233934	0.0312838	2.1426519
H	-3.5415407	0.0245472	2.1390036
H	-3.5415407	0.0245472	-2.1390036
H	-6.0233934	0.0312838	-2.1426519
C	2.1750757	0.0196118	0.0000000
C	2.8793270	0.0183088	1.1998805
C	4.2689336	0.0156448	1.2018648
C	4.9803627	0.0118583	0.0000000
C	4.2689336	0.0156448	-1.2018648
C	2.8793270	0.0183088	-1.1998805
H	1.0951632	0.0213686	0.0000000
H	2.3468410	0.0189799	2.1411192
H	4.8099569	0.0204500	2.1400300

H	4.8099569	0.0204500	-2.1400300
H	2.3468410	0.0189799	-2.1411192
Cl	-1.6850726	0.0199911	0.0000000
H	-7.2658179	0.0349010	0.0000000
N	6.3771665	0.0725400	0.0000000
H	6.8062445	-0.3029808	0.8301287
H	6.8062445	-0.3029808	-0.8301287

67.

C	0.0000000	0.0000000	5.5670641
C	1.2119079	0.0000000	4.8985947
C	1.2113125	0.0000000	3.5082011
C	0.0000000	0.0000000	2.8266885
C	-1.2113125	0.0000000	3.5082011
C	-1.2119079	0.0000000	4.8985947
H	2.1345944	0.0000000	5.4597923
H	2.1400303	0.0000000	2.9570553
H	-2.1400303	0.0000000	2.9570553
H	-2.1345944	0.0000000	5.4597923
C	0.0000000	0.0000000	-2.7567938
C	1.2046655	0.0000000	-3.4531966
C	1.2133898	0.0000000	-4.8446757
C	0.0000000	0.0000000	-5.5101224
C	-1.2133898	0.0000000	-4.8446757
C	-1.2046655	0.0000000	-3.4531966
H	0.0000000	0.0000000	-1.6769233
H	2.1420576	0.0000000	-2.9151346
H	2.1332562	0.0000000	-5.4106161
H	-2.1332562	0.0000000	-5.4106161
H	-2.1420576	0.0000000	-2.9151346
F	0.0000000	0.0000000	-6.8573187
F	0.0000000	0.0000000	6.9112759
Cl	0.0000000	0.0000000	1.0960884

68.

C	0.0000000	0.0000000	5.5574710
C	1.2118618	0.0000000	4.8889868
C	1.2111995	-0.0000000	3.4985794
C	0.0000000	0.0000000	2.8167114
C	-1.2111995	0.0000000	3.4985794
C	-1.2118618	0.0000000	4.8889868
H	2.1345702	0.0000000	5.4501436
H	2.1399172	0.0000000	2.9474141
H	-2.1399172	0.0000000	2.9474141
H	-2.1345702	0.0000000	5.4501436
C	0.0000000	0.0000000	-2.7698844
C	1.2059533	0.0000000	-3.4649106
C	1.2055928	0.0000000	-4.8568733
C	0.0000000	0.0000000	-5.5519581
C	-1.2055928	0.0000000	-4.8568733
C	-1.2059533	0.0000000	-3.4649106

H	-0.0000000	0.0000000	-1.6890183
H	2.1415589	0.0000000	-2.9226921
H	2.1405214	0.0000000	-5.4000971
H	-2.1405214	0.0000000	-5.4000971
H	-2.1415589	0.0000000	-2.9226921
H	0.0000000	0.0000000	-6.6328653
F	0.0000000	0.0000000	6.9019014
Cl	0.0000000	0.0000000	1.0865409

69.

C	-6.1461932	0.0316237	0.0000000
C	-5.4776711	0.0298486	1.2118225
C	-4.0872624	0.0263200	1.2110479
C	-3.4049536	0.0246481	0.0000000
C	-4.0872624	0.0263200	-1.2110479
C	-5.4776711	0.0298486	-1.2118225
H	-6.0388404	0.0313548	2.1345324
H	-3.5362353	0.0250277	2.1398711
H	-3.5362353	0.0250277	-2.1398711
H	-6.0388404	0.0313548	-2.1345324
C	2.1856053	0.0184876	0.0000000
C	2.8897552	0.0174338	1.1999365
C	4.2793228	0.0152330	1.2018972
C	4.9908027	0.0117608	0.0000000
C	4.2793228	0.0152330	-1.2018972
C	2.8897552	0.0174338	-1.1999365
H	1.1056888	0.0199245	0.0000000
H	2.3574092	0.0179604	2.1412572
H	4.8203048	0.0201178	2.1400673
H	4.8203048	0.0201178	-2.1400673
H	2.3574092	0.0179604	-2.1412572
Cl	-1.6751478	0.0210513	0.0000000
F	-7.4908212	0.0352887	0.0000000
N	6.3872654	0.0730276	0.0000000
H	6.8170940	-0.3012022	0.8302834
H	6.8170940	-0.3012022	-0.8302834

70.

C	-5.6144910	0.0239864	0.0000000
C	-4.9003879	0.0294230	1.2003722
C	-3.5114830	0.0343660	1.2063281
C	-2.8231659	0.0366866	0.0000000
C	-3.5114830	0.0343660	-1.2063281
C	-4.9003879	0.0294230	-1.2003722
H	-5.4360982	0.0339213	2.1412033
H	-2.9658186	0.0360280	2.1387923
H	-2.9658186	0.0360280	-2.1387923
H	-5.4360982	0.0339213	-2.1412033
C	2.7605109	0.0505538	0.0000000
C	3.4649072	0.0498652	1.1998035

C	4.8545634	0.0483275	1.2018325
C	5.5659445	0.0451843	0.0000000
C	4.8545634	0.0483275	-1.2018325
C	3.4649072	0.0498652	-1.1998035
H	1.6806065	0.0511372	0.0000000
H	2.9322691	0.0502070	2.1409573
H	5.3956309	0.0533900	2.1399921
H	5.3956309	0.0533900	-2.1399921
H	2.9322691	0.0502070	-2.1409573
Cl	-1.0911073	0.0410162	0.0000000
N	6.9630250	0.1072965	0.0000000
H	7.3918555	-0.2689560	0.8299664
H	7.3918555	-0.2689560	-0.8299664
N	-7.0089794	0.0831738	0.0000000
H	-7.4416101	-0.2860894	-0.8308543
H	-7.4416101	-0.2860894	0.8308543

71.

C	-5.0248496	0.0175629	0.0000000
C	-4.3107409	0.0211989	1.2004233
C	-2.9218522	0.0230135	1.2064664
C	-2.2339141	0.0238547	0.0000000
C	-2.9218522	0.0230135	-1.2064664
C	-4.3107409	0.0211989	-1.2004233
H	-4.8464093	0.0266086	2.1412569
H	-2.3760523	0.0233727	2.1388345
H	-2.3760523	0.0233727	-2.1388345
H	-4.8464093	0.0266086	-2.1412569
C	3.3414708	0.0251316	0.0000000
C	4.0370732	0.0225102	1.2054096
C	5.4289599	0.0173159	1.2054959
C	6.1248875	0.0146751	0.0000000
C	5.4289599	0.0173159	-1.2054959
C	4.0370732	0.0225102	-1.2054096
H	2.2607570	0.0290877	0.0000000
H	3.4951413	0.0246517	2.1410666
H	5.9698056	0.0153617	2.1418076
H	5.9698056	0.0153617	-2.1418076
H	3.4951413	0.0246517	-2.1410666
Cl	-0.5015618	0.0244792	0.0000000
N	-6.4189206	0.0803675	0.0000000
H	-6.8529448	-0.2869697	-0.8309603
H	-6.8529448	-0.2869697	0.8309603
H	7.2061699	0.0107145	0.0000000

72.

C	-5.0295499	0.0141930	0.0000000
C	-4.3153799	0.0183554	1.2004673
C	-2.9265134	0.0212259	1.2065392
C	-2.2388830	0.0225579	0.0000000
C	-2.9265134	0.0212259	-1.2065392

C	-4.3153799	0.0183554	-1.2004673
H	-4.8510101	0.0233430	2.1413018
H	-2.3809937	0.0219865	2.1390651
H	-2.3809937	0.0219865	-2.1390651
H	-4.8510101	0.0233430	-2.1413018
C	3.3243065	0.0274016	0.0000000
C	4.0209647	0.0252113	1.2045219
C	5.4125027	0.0207075	1.2133484
C	6.0779225	0.0184337	0.0000000
C	5.4125027	0.0207075	-1.2133484
C	4.0209647	0.0252113	-1.2045219
H	2.2444979	0.0303902	0.0000000
H	3.4825762	0.0270349	2.1417307
H	5.9784499	0.0189103	2.1332252
H	5.9784499	0.0189103	-2.1332252
H	3.4825762	0.0270349	-2.1417307
Cl	-0.5061351	0.0242013	0.0000000
N	-6.4232875	0.0758990	0.0000000
H	-6.8578050	-0.2902435	-0.8311737
H	-6.8578050	-0.2902435	0.8311737
F	7.4255457	0.0138608	0.0000000

73.

C	5.7251753	-0.0014811	0.0000000
C	5.0262731	-1.2045954	0.0000000
C	3.6348020	-1.2122179	0.0000000
C	2.9566698	0.0014038	0.0000000
C	3.6373336	1.2136087	0.0000000
C	5.0287849	1.2030891	0.0000000
H	6.8057771	-0.0026137	0.0000000
H	5.5613568	-2.1440196	0.0000000
H	3.0835822	-2.1412307	0.0000000
H	3.0880499	2.1437671	0.0000000
H	5.5658265	2.1413951	0.0000000
C	-2.8744512	0.0008938	0.0000000
C	-3.5682978	-1.2053533	0.0000000
C	-4.9601257	-1.2061005	0.0000000
C	-5.6566393	-0.0009750	0.0000000
C	-4.9617472	1.2050853	0.0000000
C	-3.5699184	1.2062077	0.0000000
H	-1.7936243	0.0015972	0.0000000
H	-3.0256207	-2.1405335	0.0000000
H	-5.5003768	-2.1426815	0.0000000
H	-6.7378733	-0.0017051	0.0000000
H	-5.5032566	2.1409398	0.0000000
H	-3.0285048	2.1421201	0.0000000
Br	1.0668048	0.0033995	0.0000000

74.

C	0.0000000	0.0000000	5.7361784
C	1.2038735	0.0000000	5.0385604

C	1.2130604	0.0000000	3.6470612
C	0.0000000	0.0000000	2.9680103
C	-1.2130604	0.0000000	3.6470612
C	-1.2038735	0.0000000	5.0385604
H	0.0000000	0.0000000	6.8167907
H	2.1427333	0.0000000	5.5746359
H	2.1426993	0.0000000	3.0968944
H	-2.1426993	0.0000000	3.0968944
H	-2.1427333	0.0000000	5.5746359
C	0.0000000	0.0000000	-2.8627162
C	1.2048891	0.0000000	-3.5585475
C	1.2134502	0.0000000	-4.9500261
C	0.0000000	0.0000000	-5.6154057
C	-1.2134502	0.0000000	-4.9500261
C	-1.2048891	0.0000000	-3.5585475
H	0.0000000	0.0000000	-1.7827707
H	2.1421042	0.0000000	-3.0201998
H	2.1332934	0.0000000	-5.5159902
H	-2.1332934	0.0000000	-5.5159902
H	-2.1421042	0.0000000	-3.0201998
Br	0.0000000	0.0000000	1.0777684
F	0.0000000	0.0000000	-6.9626320

75.

C	-6.3197866	0.0320585	0.0000000
C	-5.6220128	0.0302074	1.2038178
C	-4.2305451	0.0269142	1.2128017
C	-3.5506556	0.0254611	0.0000000
C	-4.2305451	0.0269142	-1.2128017
C	-5.6220128	0.0302074	-1.2038178
H	-6.1581122	0.0313687	2.1427008
H	-3.6802400	0.0257201	2.1423917
H	-3.6802400	0.0257201	-2.1423917
H	-6.1581122	0.0313687	-2.1427008
C	2.2802941	0.0194815	0.0000000
C	2.9838505	0.0180014	1.2001766
C	4.3733879	0.0148943	1.2019738
C	5.0847664	0.0108615	0.0000000
C	4.3733879	0.0148943	-1.2019738
C	2.9838505	0.0180014	-1.2001766
H	1.2003075	0.0213204	0.0000000
H	2.4511490	0.0187877	2.1412773
H	4.9144125	0.0194321	2.1401086
H	4.9144125	0.0194321	-2.1401086
H	2.4511490	0.0187877	-2.1412773
Br	-1.6610402	0.0221157	0.0000000
H	-7.4003980	0.0347408	0.0000000
N	6.4813268	0.0709743	0.0000000
H	6.9107029	-0.3038330	0.8302630
H	6.9107029	-0.3038330	-0.8302630

76.

C	0.0000000	0.0000000	5.6961945
C	1.2118724	0.0000000	5.0277696
C	1.2118236	0.0000000	3.6369119
C	0.0000000	0.0000000	2.9550468
C	-1.2118236	0.0000000	3.6369119
C	-1.2118724	0.0000000	5.0277696
H	2.1346655	0.0000000	5.5890306
H	2.1433707	0.0000000	3.0905246
H	-2.1433707	0.0000000	3.0905246
H	-2.1346655	0.0000000	5.5890306
C	0.0000000	0.0000000	-2.8740026
C	1.2049704	0.0000000	-3.5697102
C	1.2134824	0.0000000	-4.9611507
C	0.0000000	0.0000000	-5.6265269
C	-1.2134824	0.0000000	-4.9611507
C	-1.2049704	0.0000000	-3.5697102
H	0.0000000	0.0000000	-1.7940191
H	2.1422104	0.0000000	-3.0314010
H	2.1333086	0.0000000	-5.5271330
H	-2.1333086	0.0000000	-5.5271330
H	-2.1422104	0.0000000	-3.0314010
Br	0.0000000	0.0000000	1.0669522
F	0.0000000	0.0000000	-6.9735616
F	0.0000000	0.0000000	7.0402331

77.

C	0.0000000	0.0000000	5.6855407
C	1.2118467	0.0000000	5.0171358
C	1.2117167	0.0000000	3.6262755
C	0.0000000	0.0000000	2.9440675
C	-1.2117167	0.0000000	3.6262755
C	-1.2118467	0.0000000	5.0171358
H	2.1346483	0.0000000	5.5783897
H	2.1431911	0.0000000	3.0797381
H	-2.1431911	0.0000000	3.0797381
H	-2.1346483	0.0000000	5.5783897
C	0.0000000	0.0000000	-2.8860525
C	1.2058831	0.0000000	-3.5805907
C	1.2056362	0.0000000	-4.9724399
C	0.0000000	0.0000000	-5.6681038
C	-1.2056362	0.0000000	-4.9724399
C	-1.2058831	0.0000000	-3.5805907
H	0.0000000	0.0000000	-1.8051485
H	2.1415032	0.0000000	-3.0386268
H	2.1418424	0.0000000	-5.5133605
H	-2.1418424	0.0000000	-5.5133605
H	-2.1415032	0.0000000	-3.0386268
Br	0.0000000	0.0000000	1.0562130
F	0.0000000	0.0000000	7.0298014
H	0.0000000	0.0000000	-6.7493604

78.

C	-6.2802784	0.0316445	0.0000000
C	-5.6118541	0.0302398	1.2118095
C	-4.2209773	0.0273807	1.2115797
C	-3.5383521	0.0260034	0.0000000
C	-4.2209773	0.0273807	-1.2115797
C	-5.6118541	0.0302398	-1.2118095
H	-6.1731052	0.0315346	2.1346242
H	-3.6744099	0.0263538	2.1430536
H	-3.6744099	0.0263538	-2.1430536
H	-6.1731052	0.0315346	-2.1346242
C	2.2904613	0.0186883	0.0000000
C	2.9938990	0.0174243	1.2002398
C	4.3833982	0.0145046	1.2020065
C	5.0948099	0.0107093	0.0000000
C	4.3833982	0.0145046	-1.2020065
C	2.9938990	0.0174243	-1.2002398
H	1.2104559	0.0204557	0.0000000
H	2.4613256	0.0179532	2.1414128
H	4.9244018	0.0193086	2.1401390
H	4.9244018	0.0193086	-2.1401390
H	2.4613256	0.0179532	-2.1414128
Br	-1.6508192	0.0228535	0.0000000
F	-7.6247317	0.0345198	0.0000000
N	6.4910454	0.0710774	0.0000000
H	6.9210265	-0.3026756	0.8303926
H	6.9210265	-0.3026756	-0.8303926

79.

C	-5.7432951	0.0235306	0.0000000
C	-5.0291552	0.0289391	1.2003453
C	-3.6399203	0.0339533	1.2069252
C	-2.9513055	0.0361411	0.0000000
C	-3.6399203	0.0339533	-1.2069252
C	-5.0291552	0.0289391	-1.2003453
H	-5.5649062	0.0331272	2.1413080
H	-3.0986525	0.0356938	2.1419619
H	-3.0986525	0.0356938	-2.1419619
H	-5.5649062	0.0331272	-2.1413080
C	2.8792038	0.0498399	0.0000000
C	3.5829068	0.0495476	1.2001000
C	4.9724977	0.0486974	1.2019395
C	5.6838284	0.0457465	0.0000000
C	4.9724977	0.0486974	-1.2019395
C	3.5829068	0.0495476	-1.2001000
H	1.7992104	0.0498071	0.0000000
H	3.0500699	0.0495769	2.1411259
H	5.5135624	0.0542239	2.1400693
H	5.5135624	0.0542239	-2.1400693
H	3.0500699	0.0495769	-2.1411259

Br	-1.0622709	0.0403884	0.0000000
N	7.0806477	0.1080055	0.0000000
H	7.5099441	-0.2673746	0.8300914
H	7.5099441	-0.2673746	-0.8300914
N	-7.1371519	0.0826168	0.0000000
H	-7.5707800	-0.2844227	-0.8312756
H	-7.5707800	-0.2844227	0.8312756

80.

C	-5.1483782	0.0133353	0.0000000
C	-4.4342138	0.0178284	1.2003897
C	-3.0450089	0.0212711	1.2070603
C	-2.3567890	0.0228760	0.0000000
C	-3.0450089	0.0212711	-1.2070603
C	-4.4342138	0.0178284	-1.2003897
H	-4.9698985	0.0224532	2.1413718
H	-2.5037783	0.0220990	2.1421064
H	-2.5037783	0.0220990	-2.1421064
H	-4.9698985	0.0224532	-2.1413718
C	3.4736392	0.0279725	0.0000000
C	4.1685308	0.0256675	1.2057318
C	5.5603549	0.0210777	1.2055855
C	6.2561861	0.0187032	0.0000000
C	5.5603549	0.0210777	-1.2055855
C	4.1685308	0.0256675	-1.2057318
H	2.3928051	0.0311353	0.0000000
H	3.6263013	0.0274801	2.1411984
H	6.1014235	0.0193359	2.1417653
H	6.1014235	0.0193359	-2.1417653
H	3.6263013	0.0274801	-2.1411984
Br	-0.4674786	0.0247937	0.0000000
N	-6.5418366	0.0741230	0.0000000
H	-6.9765476	-0.2912436	-0.8314053
H	-6.9765476	-0.2912436	0.8314053
H	7.3375249	0.0151226	0.0000000

81.

C	-5.1583473	0.0135580	0.0000000
C	-4.4441432	0.0177426	1.2004381
C	-3.0549557	0.0206349	1.2071719
C	-2.3670839	0.0219272	0.0000000
C	-3.0549557	0.0206349	-1.2071719
C	-4.4441432	0.0177426	-1.2004381
H	-4.9797919	0.0226274	2.1414178
H	-2.5137808	0.0213413	2.1422359
H	-2.5137808	0.0213413	-2.1422359
H	-4.9797919	0.0226274	-2.1414178
C	3.4626509	0.0260901	0.0000000
C	4.1586305	0.0247184	1.2048200
C	5.5501303	0.0219993	1.2134416
C	6.2154700	0.0206389	0.0000000

C	5.5501303	0.0219993	-1.2134416
C	4.1586305	0.0247184	-1.2048200
H	2.3827292	0.0283552	0.0000000
H	3.6200426	0.0258791	2.1419043
H	6.1161413	0.0209792	2.1332679
H	6.1161413	0.0209792	-2.1332679
H	3.6200426	0.0258791	-2.1419043
Br	-0.4775236	0.0230428	0.0000000
N	-6.5514545	0.0748392	0.0000000
H	-6.9869550	-0.2891275	-0.8315678
H	-6.9869550	-0.2891275	0.8315678
F	7.5629234	0.0179591	0.0000000

82.

C	0.0000000	0.0000000	5.8129517
C	1.2030131	0.0000000	5.1145492
C	1.2106156	0.0000000	3.7231644
C	0.0000000	0.0000000	3.0361570
C	-1.2106156	0.0000000	3.7231644
C	-1.2030131	0.0000000	5.1145492
H	2.1424677	0.0000000	5.6496945
H	2.1440621	0.0000000	3.1788029
H	-2.1440621	0.0000000	3.1788029
H	-2.1424677	0.0000000	5.6496945
C	0.0000000	0.0000000	-2.9479278
C	1.2063251	0.0000000	-3.6412646
C	1.2058183	0.0000000	-5.0330643
C	0.0000000	0.0000000	-5.7284959
C	-1.2058183	0.0000000	-5.0330643
C	-1.2063251	0.0000000	-3.6412646
H	0.0000000	0.0000000	-1.8671916
H	2.1414074	0.0000000	-3.0983389
H	2.1419369	0.0000000	-5.5741189
H	-2.1419369	0.0000000	-5.5741189
H	-2.1414074	0.0000000	-3.0983389
H	0.0000000	0.0000000	-6.8097226
H	0.0000000	0.0000000	6.8936398
I	0.0000000	0.0000000	0.9717406

83.

C	0.0000000	0.0000000	5.8244939
C	1.2030436	0.0000000	5.1261648
C	1.2107812	0.0000000	3.7347757
C	0.0000000	0.0000000	3.0482316
C	-1.2107812	0.0000000	3.7347757
C	-1.2030436	0.0000000	5.1261648
H	2.1424601	0.0000000	5.6613329
H	2.1442209	0.0000000	3.1904487
H	-2.1442209	0.0000000	3.1904487
H	-2.1424601	0.0000000	5.6613329
C	0.0000000	0.0000000	-2.9368227

C	1.2053776	0.0000000	-3.6313956
C	1.2136810	0.0000000	-5.0227773
C	0.0000000	0.0000000	-5.6879263
C	-1.2136810	0.0000000	-5.0227773
C	-1.2053776	0.0000000	-3.6313956
H	0.0000000	0.0000000	-1.8570303
H	2.1420648	0.0000000	-3.0920877
H	2.1334069	0.0000000	-5.5888898
H	-2.1334069	0.0000000	-5.5888898
H	-2.1420648	0.0000000	-3.0920877
F	0.0000000	0.0000000	-7.0349315
H	0.0000000	0.0000000	6.9051513
I	0.0000000	0.0000000	0.9836910

84.

C	-6.4239622	0.0325245	0.0000000
C	-5.7254597	0.0306790	1.2029575
C	-4.3340785	0.0270017	1.2104609
C	-3.6465801	0.0252203	0.0000000
C	-4.3340785	0.0270017	-1.2104609
C	-5.7254597	0.0306790	-1.2029575
H	-6.2605141	0.0323148	2.1424776
H	-3.7896911	0.0257644	2.1439042
H	-3.7896911	0.0257644	-2.1439042
H	-6.2605141	0.0323148	-2.1424776
C	2.3575019	0.0204697	0.0000000
C	3.0598562	0.0183886	1.2006336
C	4.4492855	0.0145086	1.2022005
C	5.1604865	0.0098712	0.0000000
C	4.4492855	0.0145086	-1.2022005
C	3.0598562	0.0183886	-1.2006336
H	1.2777157	0.0228734	0.0000000
H	2.5263651	0.0195644	2.1412982
H	4.9903726	0.0186016	2.1402542
H	4.9903726	0.0186016	-2.1402542
H	2.5263651	0.0195644	-2.1412982
I	-1.5823332	0.0210353	0.0000000
H	-7.5046498	0.0353280	0.0000000
N	6.5567010	0.0689634	0.0000000
H	6.9864242	-0.3049661	0.8304330
H	6.9864242	-0.3049661	-0.8304330

85.

C	0.0000000	0.0000000	5.7832993
C	1.2111111	0.0000000	5.1143276
C	1.2095935	0.0000000	3.7235440
C	0.0000000	0.0000000	3.0344283
C	-1.2095935	0.0000000	3.7235440
C	-1.2111111	0.0000000	5.1143276
H	2.1344497	0.0000000	5.6747577
H	2.1448003	0.0000000	3.1828532

H	-2.1448003	0.0000000	3.1828532
H	-2.1344497	0.0000000	5.6747577
C	0.0000000	0.0000000	-2.9470339
C	1.2054643	0.0000000	-3.6414326
C	1.2137076	0.0000000	-5.0327816
C	0.0000000	0.0000000	-5.6979385
C	-1.2137076	0.0000000	-5.0327816
C	-1.2054643	0.0000000	-3.6414326
H	0.0000000	0.0000000	-1.8672324
H	2.1422113	0.0000000	-3.1022334
H	2.1334199	0.0000000	-5.5989041
H	-2.1334199	0.0000000	-5.5989041
H	-2.1422113	0.0000000	-3.1022334
F	0.0000000	0.0000000	-7.0447302
F	0.0000000	0.0000000	7.1271271
I	0.0000000	0.0000000	0.9718188

86.

C	0.0000000	0.0000000	5.7699974
C	1.2110661	0.0000000	5.1009982
C	1.2094367	0.0000000	3.7101945
C	0.0000000	0.0000000	3.0206454
C	-1.2094367	0.0000000	3.7101945
C	-1.2110661	0.0000000	5.1009982
H	2.1344339	0.0000000	5.6613922
H	2.1446402	0.0000000	3.1694673
H	-2.1446402	0.0000000	3.1694673
H	-2.1344339	0.0000000	5.6613922
C	0.0000000	0.0000000	-2.9564211
C	1.2064211	0.0000000	-3.6495585
C	1.2058410	0.0000000	-5.0413440
C	0.0000000	0.0000000	-5.7367204
C	-1.2058410	0.0000000	-5.0413440
C	-1.2064211	0.0000000	-3.6495585
H	0.0000000	0.0000000	-1.8756697
H	2.1415654	0.0000000	-3.1067600
H	2.1419263	0.0000000	-5.5824146
H	-2.1419263	0.0000000	-5.5824146
H	-2.1415654	0.0000000	-3.1067600
H	0.0000000	0.0000000	-6.8179232
F	0.0000000	0.0000000	7.1140373
I	0.0000000	0.0000000	0.9581041

87.

C	-6.3841386	0.0326036	0.0000000
C	-5.7151362	0.0308080	1.2110139
C	-4.3242972	0.0273298	1.2093049
C	-3.6343509	0.0256634	0.0000000
C	-4.3242972	0.0273298	-1.2093049
C	-5.7151362	0.0308080	-1.2110139
H	-6.2754097	0.0322600	2.1344668

H	-3.7834868	0.0260292	2.1444631
H	-3.7834868	0.0260292	-2.1444631
H	-6.2754097	0.0322600	-2.1344668
C	2.3675088	0.0194811	0.0000000
C	3.0696927	0.0175262	1.2007140
C	4.4590791	0.0140673	1.2022402
C	5.1703198	0.0097185	0.0000000
C	4.4590791	0.0140673	-1.2022402
C	3.0696927	0.0175262	-1.2007140
H	1.2877376	0.0217150	0.0000000
H	2.5363080	0.0183949	2.1414358
H	5.0001335	0.0182655	2.1402894
H	5.0001335	0.0182655	-2.1402894
H	2.5363080	0.0183949	-2.1414358
I	-1.5720255	0.0218224	0.0000000
F	-7.7283799	0.0362916	0.0000000
N	6.5661884	0.0695236	0.0000000
H	6.9966869	-0.3030906	0.8305868
H	6.9966869	-0.3030906	-0.8305868

88.

C	-5.8392088	0.0225900	0.0000000
C	-5.1243887	0.0278576	1.1995684
C	-3.7354797	0.0336412	1.2046493
C	-3.0393776	0.0367994	0.0000000
C	-3.7354797	0.0336412	-1.2046493
C	-5.1243887	0.0278576	-1.1995684
H	-5.6592522	0.0313055	2.1410663
H	-3.1999523	0.0354314	2.1433780
H	-3.1999523	0.0354314	-2.1433780
H	-5.6592522	0.0313055	-2.1410663
C	2.9632507	0.0503949	0.0000000
C	3.6657250	0.0499468	1.2005532
C	5.0551884	0.0487877	1.2021817
C	5.7663532	0.0455814	0.0000000
C	5.0551884	0.0487877	-1.2021817
C	3.6657250	0.0499468	-1.2005532
H	1.8835279	0.0501738	0.0000000
H	3.1320658	0.0501853	2.1411284
H	5.5963403	0.0541784	2.1402148
H	5.5963403	0.0541784	-2.1402148
H	3.1320658	0.0501853	-2.1411284
I	-0.9767149	0.0417046	0.0000000
N	7.1627959	0.1072814	0.0000000
H	7.5926229	-0.2669217	0.8302957
H	7.5926229	-0.2669217	-0.8302957
N	-7.2320857	0.0815621	0.0000000
H	-7.6671400	-0.2824560	-0.8317666
H	-7.6671400	-0.2824560	0.8317666

89.

C	-5.2363733	-0.0000196	0.0000000
C	-4.5215261	0.0064459	1.1996209
C	-3.1326568	0.0144132	1.2048076
C	-2.4370179	0.0182806	0.0000000
C	-3.1326568	0.0144132	-1.2048076
C	-4.5215261	0.0064459	-1.1996209
H	-5.0564062	0.0093727	2.1410946
H	-2.5971045	0.0171067	2.1435004
H	-2.5971045	0.0171067	-2.1435004
H	-5.0564062	0.0093727	-2.1410946
C	3.5621854	0.0328743	0.0000000
C	4.2557559	0.0336168	1.2061890
C	5.6476498	0.0355716	1.2058044
C	6.3430436	0.0366381	0.0000000
C	5.6476498	0.0355716	-1.2058044
C	4.2557559	0.0336168	-1.2061890
H	2.4815161	0.0322558	0.0000000
H	3.7128609	0.0328296	2.1412961
H	6.1883707	0.0365065	2.1421034
H	6.1883707	0.0365065	-2.1421034
H	3.7128609	0.0328296	-2.1412961
I	-0.3744051	0.0277731	0.0000000
H	7.4241835	0.0386391	0.0000000
N	-6.6289891	0.0562918	0.0000000
H	-7.0640151	-0.3072297	-0.8319554
H	-7.0640151	-0.3072297	0.8319554

90.

C	-5.2473826	-0.0000350	0.0000000
C	-4.5324985	0.0058821	1.1996724
C	-3.1436569	0.0129575	1.2049484
C	-2.4484170	0.0164664	0.0000000
C	-3.1436569	0.0129575	-1.2049484
C	-4.5324985	0.0058821	-1.1996724
H	-5.0673293	0.0090391	2.1411498
H	-2.6081504	0.0151582	2.1436506
H	-2.6081504	0.0151582	-2.1436506
H	-5.0673293	0.0090391	-2.1411498
C	3.5523259	0.0338444	0.0000000
C	4.2471276	0.0345074	1.2052593
C	5.6385505	0.0359812	1.2136396
C	6.3037053	0.0366826	0.0000000
C	5.6385505	0.0359812	-1.2136396
C	4.2471276	0.0345074	-1.2052593
H	2.4725982	0.0329794	0.0000000
H	3.7077723	0.0340741	2.1419212
H	6.2045829	0.0366782	2.1334300
H	6.2045829	0.0366782	-2.1334300
H	3.7077723	0.0340741	-2.1419212
I	-0.3857432	0.0246110	0.0000000
F	7.6509557	0.0381895	0.0000000

N	-6.6396031	0.0574489	0.0000000
H	-7.0756178	-0.3043716	-0.8321372
H	-7.0756178	-0.3043716	0.8321372

91.

C	2.8206823	-0.0750443	0.0000000
H	3.1773677	-0.5895267	0.8857257
H	1.7360957	-0.0657809	0.0000000
H	3.1773677	-0.5895267	-0.8857257
C	-2.9700668	0.0766686	0.0000000
H	-3.3068508	0.5939482	-0.8894792
H	-3.3068508	0.5939482	0.8894792
Cl	-1.1946389	0.0573668	0.0000000
H	3.1906920	0.9445310	0.0000000
H	-3.3237979	-0.9465842	0.0000000

92.

C	2.7335228	-0.1045245	0.0000000
H	3.0909064	-0.6121922	0.8906188
H	1.6489912	-0.0675998	0.0000000
H	3.0909064	-0.6121922	-0.8906188
C	-2.9082565	0.0439083	0.0000000
H	-3.2390491	0.5642884	-0.8898000
H	-3.2390491	0.5642884	0.8898000
Cl	-1.1327187	0.0091680	0.0000000
F	3.2239583	1.1914692	0.0000000
H	-3.2692117	-0.9766137	0.0000000

93.

C	2.1021984	-0.3367580	0.0000000
H	2.4319282	-0.8908189	0.8761796
H	1.0147376	-0.3350784	0.0000000
H	2.4319282	-0.8908189	-0.8761796
C	-3.5457098	-0.1986789	0.0000000
H	-3.8784207	0.3214178	-0.8894848
H	-3.8784207	0.3214178	0.8894848
Cl	-1.7709298	-0.2314540	0.0000000
N	2.6172788	1.0361283	0.0000000
H	3.1916002	1.2118257	0.8088866
H	3.1916002	1.2118257	-0.8088866
H	-3.9077906	-1.2190081	0.0000000

94.

C	2.7436763	-0.0774615	0.0000000
H	3.1003524	-0.5848598	0.8909461
H	1.6591240	-0.0420372	0.0000000
H	3.1003524	-0.5848598	-0.8909461
C	-2.8929607	0.0712334	0.0000000
H	-3.2190688	0.5754181	-0.9019278
H	-3.2190688	0.5754181	0.9019278

Cl	-1.1287076	0.0493369	0.0000000
F	-3.3754402	-1.2013248	0.0000000
F	3.2317411	1.2191366	0.0000000

95.

C	2.8312591	-0.0474708	0.0000000
H	3.1872149	-0.5622332	0.8857698
H	1.7467287	-0.0385693	0.0000000
H	3.1872149	-0.5622332	-0.8857698
C	-2.9557236	0.1042214	0.0000000
H	-3.2877683	0.6051161	-0.9015908
H	-3.2877683	0.6051161	0.9015908
Cl	-1.1923766	0.0963259	0.0000000
F	-3.4299719	-1.1723385	0.0000000
H	3.2011910	0.9720656	0.0000000

96.

C	2.1109687	-0.3138262	0.0000000
H	2.4393663	-0.8679998	0.8764000
H	1.0234208	-0.3130728	0.0000000
H	2.4393663	-0.8679998	-0.8764000
C	-3.5314646	-0.1756685	0.0000000
H	-3.8599446	0.3278706	-0.9014884
H	-3.8599446	0.3278706	0.9014884
Cl	-1.7693696	-0.1963732	0.0000000
F	-4.0166133	-1.4489228	0.0000000
N	2.6251160	1.0590544	0.0000000
H	3.1995492	1.2345338	0.8089152
H	3.1995492	1.2345338	-0.8089152

97.

C	2.7901055	-0.0553049	0.0000000
H	3.1142216	-0.6130101	0.8760973
H	1.7024241	-0.0394392	0.0000000
H	3.1142216	-0.6130101	-0.8760973
C	-3.0247001	0.1119247	0.0000000
H	-3.1980214	0.7043033	-0.8896309
H	-3.1980214	0.7043033	0.8896309
Cl	-1.2210017	-0.2030557	0.0000000
N	-3.8338214	-1.0432211	0.0000000
H	-3.6843923	-1.6116812	0.8204624
H	-3.6843923	-1.6116812	-0.8204624
N	3.3231848	1.3112916	0.0000000
H	3.9000963	1.4792903	0.8087073
H	3.9000963	1.4792903	-0.8087073

98.

C	3.4667723	0.2128853	0.0000000
H	3.8287434	-0.2982680	0.8856146
H	2.3819992	0.2140220	0.0000000

H	3.8287434	-0.2982680	-0.8856146
C	-2.3617210	0.3771303	0.0000000
H	-2.5131836	0.9750648	-0.8898714
H	-2.5131836	0.9750648	0.8898714
Cl	-0.5689884	-0.0015963	0.0000000
H	3.8283766	1.2355791	0.0000000
N	-3.2104442	-0.7482808	0.0000000
H	-3.0835570	-1.3216667	0.8206743
H	-3.0835570	-1.3216667	-0.8206743

99.

C	3.4195494	0.1765704	0.0000000
H	3.7631483	-0.3412129	0.8903719
H	2.3362883	0.2468277	0.0000000
H	3.7631483	-0.3412129	-0.8903719
C	-2.3617979	0.3608328	0.0000000
H	-2.5265173	0.9549517	-0.8899770
H	-2.5265173	0.9549517	0.8899770
Cl	-0.5562388	0.0284399	0.0000000
N	-3.1782326	-0.7860463	0.0000000
H	-3.0413052	-1.3558068	0.8213205
H	-3.0413052	-1.3558068	-0.8213205
F	3.9497800	1.4575117	0.0000000

100.

C	2.9588046	-0.0780715	0.0000000
H	3.3082453	-0.5971954	0.8858490
H	1.8742102	-0.0548425	0.0000000
H	3.3082453	-0.5971954	-0.8858490
C	-3.1439214	0.0815174	0.0000000
H	-3.4683770	0.6022033	-0.8913275
H	-3.4683770	0.6022033	0.8913275
H	-3.4969875	-0.9413229	0.0000000
Br	-1.2132253	0.0459313	0.0000000
H	3.3413827	0.9367723	0.0000000

101.

C	2.9457221	-0.1119771	0.0000000
H	3.3028518	-0.6198013	0.8906343
H	1.8605056	-0.0751824	0.0000000
H	3.3028518	-0.6198013	-0.8906343
C	-3.1559274	0.0518845	0.0000000
H	-3.4792537	0.5728368	-0.8915819
H	-3.4792537	0.5728368	0.8915819
F	3.4357158	1.1835655	0.0000000
H	-3.5083374	-0.9711071	0.0000000
Br	-1.2248748	0.0167457	0.0000000

102.

C	2.2765191	-0.3426057	0.0000000
H	2.6054016	-0.8971966	0.8761256

H	1.1881711	-0.3400675	0.0000000
H	2.6054016	-0.8971966	-0.8761256
C	-3.8260484	-0.1896772	0.0000000
H	-4.1517067	0.3307519	-0.8911664
H	-4.1517067	0.3307519	0.8911664
N	2.7931297	1.0292292	0.0000000
H	3.3672682	1.2048907	0.8090079
H	3.3672682	1.2048907	-0.8090079
Br	-1.8950437	-0.2210549	0.0000000
H	-4.1786540	-1.2127159	0.0000000

103.

C	2.9570108	-0.0849758	0.0000000
H	3.3139638	-0.5925931	0.8907255
H	1.8716885	-0.0492778	0.0000000
H	3.3139638	-0.5925931	-0.8907255
C	-3.1422346	0.0757349	0.0000000
H	-3.4599648	0.5834112	-0.9024327
H	-3.4599648	0.5834112	0.9024327
F	-3.6250147	-1.1959247	0.0000000
F	3.4447272	1.2111837	0.0000000
Br	-1.2141752	0.0616236	0.0000000

104.

C	2.9700148	-0.0508661	0.0000000
H	3.3194857	-0.5698789	0.8858493
H	1.8853676	-0.0292782	0.0000000
H	3.3194857	-0.5698789	-0.8858493
C	-3.1299979	0.1059021	0.0000000
H	-3.4494034	0.6131188	-0.9021540
H	-3.4494034	0.6131188	0.9021540
F	-3.6138720	-1.1662484	0.0000000
H	3.3513755	0.9644733	0.0000000
Br	-1.2030526	0.0895375	0.0000000

105.

C	2.2866588	-0.3198695	0.0000000
H	2.6148011	-0.8744865	0.8762592
H	1.1982196	-0.3182100	0.0000000
H	2.6148011	-0.8744865	-0.8762592
C	-3.8139726	-0.1699243	0.0000000
H	-4.1340032	0.3370301	-0.9021134
H	-4.1340032	0.3370301	0.9021134
F	-4.2992921	-1.4422460	0.0000000
N	2.8019523	1.0521823	0.0000000
H	3.3761219	1.2279019	0.8090531
H	3.3761219	1.2279019	-0.8090531
Br	-1.8874057	-0.1828234	0.0000000

106.

C	2.9342416	-0.0641509	0.0000000
H	3.2583541	-0.6220311	0.8759512
H	1.8461940	-0.0494325	0.0000000
H	3.2583541	-0.6220311	-0.8759512
C	-3.1963741	0.1212559	0.0000000
H	-3.3576438	0.7135530	-0.8912579
H	-3.3576438	0.7135530	0.8912579
N	-3.9976179	-1.0345939	0.0000000
H	-3.8568669	-1.6033611	0.8216119
H	-3.8568669	-1.6033611	-0.8216119
N	3.4647862	1.3029150	0.0000000
H	4.0405579	1.4729019	0.8090492
H	4.0405579	1.4729019	-0.8090492
Br	-1.2200326	-0.1981189	0.0000000

107.

C	3.6704704	0.1932316	0.0000000
H	4.0231520	-0.3242189	0.8856606
H	2.5856965	0.2129235	0.0000000
H	4.0231520	-0.3242189	-0.8856606
C	-2.5544216	0.3960083	0.0000000
H	-2.7145552	0.9882425	-0.8915205
H	-2.7145552	0.9882425	0.8915205
H	4.0492707	1.2096584	0.0000000
N	-3.3553467	-0.7589085	0.0000000
H	-3.2181399	-1.3277630	0.8220082
H	-3.2181399	-1.3277630	-0.8220082
Br	-0.5765830	0.0745653	0.0000000

108.

C	3.6039598	0.1675120	0.0000000
H	3.9502350	-0.3483321	0.8904064
H	2.5200052	0.2304405	0.0000000
H	3.9502350	-0.3483321	-0.8904064
C	-2.5268962	0.3691349	0.0000000
H	-2.6833020	0.9611683	-0.8921322
H	-2.6833020	0.9611683	0.8921322
N	-3.3254455	-0.7850428	0.0000000
H	-3.1927835	-1.3532407	0.8229984
H	-3.1927835	-1.3532407	-0.8229984
F	4.1253041	1.4515981	0.0000000
Br	-0.5452264	0.0471665	0.0000000

109.

C	3.0017170	-0.0769684	0.0000000
H	3.3553002	-0.6042105	0.8992468
H	1.9007112	-0.0517406	0.0000000
H	3.3553002	-0.6042105	-0.8992468
C	-3.2260153	0.0864974	0.0000000
H	-3.5338393	0.6244714	-0.9073811
H	-3.5338393	0.6244714	0.9073811

H	3.3902878	0.9529060	0.0000000
I	-1.1072074	-0.0053035	0.0000000
H	-3.6024151	-0.9459126	0.0000000

110.

C	3.2998348	-0.6448824	0.0000000
H	3.5577051	-1.2249135	0.9015942
H	2.2188970	-0.4310143	0.0000000
H	3.5577051	-1.2249135	-0.9015942
C	-2.6821994	0.2217526	0.0000000
H	-3.0041938	0.7509137	-0.9076711
H	-3.0041938	0.7509137	0.9076711
F	4.0047390	0.5355899	0.0000000
H	-3.0282494	-0.8211541	0.0000000
I	-0.5617085	0.1906019	0.0000000

111.

C	3.2185242	-0.6086363	0.0000000
H	3.4330531	-1.2288037	0.8885961
H	2.1324848	-0.4075981	0.0000000
H	3.4330531	-1.2288037	-0.8885961
C	-2.6718784	0.2837195	0.0000000
H	-2.9541352	0.8362785	-0.9070921
H	-2.9541352	0.8362785	0.9070921
N	3.9843692	0.6334929	0.0000000
H	4.5786147	0.7155011	0.8218397
H	4.5786147	0.7155011	-0.8218397
I	-0.5598135	0.0877993	0.0000000
H	-3.1001073	-0.7283688	0.0000000

112.

C	3.4321015	-0.8270122	0.0000000
H	3.6374106	-1.4275273	0.9016259
H	2.3741492	-0.5183478	0.0000000
H	3.6374106	-1.4275273	-0.9016259
C	-2.4698906	0.5397134	0.0000000
H	-2.7362016	1.0876826	-0.9148467
H	-2.7362016	1.0876826	0.9148467
F	-3.0529095	-0.6878883	0.0000000
F	4.2377495	0.2868472	0.0000000
I	-0.3543162	0.3393934	0.0000000

113.

C	3.0125466	-0.0499595	0.0000000
H	3.3659024	-0.5772513	0.8992771
H	1.9115381	-0.0252713	0.0000000
H	3.3659024	-0.5772513	-0.8992771
C	-3.2141302	0.1039717	0.0000000
H	-3.5155997	0.6339389	-0.9145019
H	-3.5155997	0.6339389	0.9145019

F	-3.7206447	-1.1578014	0.0000000
H	3.4010581	0.9799591	0.0000000
I	-1.0909735	0.0357262	0.0000000

114.

C	3.6031500	-0.7834635	0.0000000
H	3.8116212	-1.4055769	0.8885865
H	2.5187297	-0.5733142	0.0000000
H	3.8116212	-1.4055769	-0.8885865
C	-2.2144452	0.4691557	0.0000000
H	-2.4432814	1.0354003	-0.9139721
H	-2.4432814	1.0354003	0.9139721
F	-2.8904712	-0.7123329	0.0000000
N	4.3800418	0.4511798	0.0000000
H	4.9743393	0.5291514	0.8221763
H	4.9743393	0.5291514	-0.8221763
I	-0.1212119	0.1137877	0.0000000

115.

C	3.5087371	-0.8110767	0.0000000
H	3.7209851	-1.4324421	0.8885405
H	2.4247960	-0.6002979	0.0000000
H	3.7209851	-1.4324421	-0.8885405
C	-2.4497719	0.5323221	0.0000000
H	-2.5704137	1.1422236	-0.9051664
H	-2.5704137	1.1422236	0.9051664
N	-3.2824751	-0.5969976	0.0000000
H	-3.1676126	-1.1744641	0.8339274
H	-3.1676126	-1.1744641	-0.8339274
N	4.2843727	0.4263069	0.0000000
H	4.8796575	0.5030750	0.8216442
H	4.8796575	0.5030750	-0.8216442
I	-0.2776042	0.0941891	0.0000000

116.

C	3.7195376	0.1746248	0.0000000
H	4.1048235	-0.3307830	0.8989899
H	2.6189253	0.1371777	0.0000000
H	4.1048235	-0.3307830	-0.8989899
C	-2.6320331	0.4130690	0.0000000
H	-2.7720806	1.0169759	-0.9061231
H	-2.7720806	1.0169759	0.9061231
H	4.0481033	1.2253847	0.0000000
N	-3.4148316	-0.7474815	0.0000000
H	-3.2839987	-1.3195768	0.8351549
H	-3.2839987	-1.3195768	-0.8351549
I	-0.4371899	0.0639931	0.0000000

117.

C	3.4807454	-0.8198930	0.0000000
H	3.7299205	-1.4042278	0.9014880

H	2.4044871	-0.5837208	0.0000000
H	3.7299205	-1.4042278	-0.9014880
C	-2.5663044	0.5363407	0.0000000
H	-2.6746236	1.1457105	-0.9067509
H	-2.6746236	1.1457105	0.9067509
N	-3.4004889	-0.5852578	0.0000000
H	-3.3045727	-1.1620791	0.8363628
H	-3.3045727	-1.1620791	-0.8363628
F	4.2063725	0.3488257	0.0000000
I	-0.3848940	0.0868140	0.0000000
